# *scribble *mutants promote aPKC and JNK-dependent epithelial neoplasia independently of Crumbs

**DOI:** 10.1186/1741-7007-7-62

**Published:** 2009-09-24

**Authors:** Gregory R Leong, Karen R Goulding, Nancy Amin, Helena E Richardson, Anthony M Brumby

**Affiliations:** 1 Peter MacCallum Cancer Centre, 7 St Andrews Place, East Melbourne, 3002, Victoria, Australia; 2 Baker IDI Heart and Diabetes Institute, 75 Commercial Road, Melbourne, 3004, Victoria, Australia; 3 Anatomy and Cell Biology Department, University of Melbourne, Melbourne, 3010, Victoria, Australia

## Abstract

**Background:**

Metastatic neoplasias are characterized by excessive cell proliferation and disruptions to apico-basal cell polarity and tissue architecture. Understanding how alterations in cell polarity can impact upon tumour development is, therefore, a central issue in cancer biology. The *Drosophila *gene *scribble *(*scrib*) encodes a PDZ-domain scaffolding protein that regulates cell polarity and acts as a tumour suppressor in flies. Increasing evidence also implicates the loss of human Scrib in cancer. In this report, we investigate how loss of Scrib promotes epithelial tumourigenesis in *Drosophila*, both alone and in cooperation with oncogenic mutations.

**Results:**

We find that genetically distinct atypical protein kinase C (aPKC)-dependent and Jun N-terminal kinase (JNK)-dependent alterations in *scrib *mutants drive epithelial tumourigenesis. First, we show that over-expression of the apical cell polarity determinants Crumbs (Crb) or aPKC induces similar cell morphology defects and over-proliferation phenotypes as *scrib *loss-of-function. However, the morphological and proliferative defects in *scrib *mutants are independent of Crb function, and instead can be rescued by a dominant negative (kinase dead) aPKC transgene. Secondly, we demonstrate that loss of Scrib promotes oncogene-mediated transformation through both aPKC and JNK-dependent pathways. JNK normally promotes apoptosis of *scrib *mutant cells. However, in cooperation with oncogenic activated Ras or Notch signalling, JNK becomes an essential driver of tumour overgrowth and invasion. aPKC-dependent signalling in *scrib *mutants cooperates with JNK to significantly enhance oncogene-mediated tumour overgrowth.

**Conclusion:**

These results demonstrate distinct aPKC and JNK-dependent pathways through which loss of Scrib promotes tumourigenesis in *Drosophila*. This is likely to have a direct relevance to the way in which human Scrib can similarly restrain an oncogene-mediated transformation and, more generally, on how the outcome of oncogenic signalling can be profoundly perturbed by defects in apico-basal epithelial cell polarity.

## Background

Metastatic cancers are associated with excessive cell proliferation and alterations to tissue architecture and tumour cell polarity. How tissue architecture and cell polarity are linked and coordinated with cell proliferation control, and how alterations in cell morphology can impact upon the outcome of oncogenic signalling pathways, are now central questions in cancer biology. In *Drosophila*, Scribble (Scrib), Discs large (Dlg) and Lethal giant larvae (Lgl), cooperatively establish and maintain apico-basal cell polarity and repress inappropriate cell proliferation and neoplasia (invasive overgrowth with a failure to differentiate) in both epithelial and neuronal tissues [1]. Furthermore, in a fly 'two-hit' model of tumourigenesis, the loss of any one of these three genes has also been shown to cooperate with oncogenic alleles of Ras resulting in neoplasia [2,3]. As the function of this group of proteins is conserved in humans (including human Scrib's ability to cooperate with oncogenes in promoting tumourigenesis [4,5]) a deeper understanding is needed of the way in which these genes function to repress neoplasia. *Drosophila*, a powerful model organism, can be used to investigate these questions as pathways regulating cell proliferation, survival, differentiation and tumour cell invasion are all highly conserved between flies and humans [reviewed in [6]].

In *Drosophila*, homozygous *scrib*, *dlg *or *lgl *mutants develop to the third instar larval stage but fail to pupate and die as overgrown larvae. Some of the mono-layered epithelial imaginal discs, notably the wing discs, become multi-layered, fail to differentiate and over-proliferate throughout the extended larval stage of development. These overgrown masses of tissue exhibit characteristics of human cancers, including a failure to cease proliferation and differentiate, loss of tissue structure and a propensity to fuse and invade the surrounding tissues. Using clonal analysis in the eye imaginal disc we have previously examined *scrib *mutant clones and shown that, although loss of Scrib is associated with altered cell morphology (indicative of aberrant cell polarity), ectopic expression of Cyclin E (CycE) and excessive cell proliferation, the mutant clones of tissue do not become overgrown because they are removed by Jun N-terminal kinase (JNK)-dependent apoptosis [2]. If, however, activated oncogenic alleles of either the small GTPase Ras (*dRas1*^*V*12 ^or shortened to Ras^ACT^) or the receptor/transcription regulator Notch (*N*^*intra *^or shortened to N^ACT^) are specifically expressed within the mutant tissue, tumours are formed which become massively overgrown throughout an extended larval stage of development and which then invade the adjacent brain and ventral nerve cord [2,3].

Most of what is known of the way in which Scrib represses epithelial neoplasia in *Drosophila *has focused on how Scrib regulates cell polarity, particularly in the embryonic ectoderm [reviewed in [7]]. Genetic analysis suggests that Scrib, in cooperation with Dlg and Lgl, promotes basolateral membrane identity and functions antagonistically towards two other protein complexes, the Crumbs (Crb) complex and the Bazooka (Baz) complex, both functioning to promote apical cell identity [8,9]. The Crb complex, incorporating Crb, Stardust (Sdt) and Patj, is anchored apically through Crb's transmembrane domain. The Baz complex is also apically enriched and can include Cdc42, atypical protein kinase C (aPKC) and Par6. Although a mechanistic understanding of how Scrib and the Crb or Baz complexes act antagonistically towards one another is still incomplete, aPKC directly phosphorylates Lgl resulting in its inactivation and the binding of Lgl to aPKC has the potential to repress the ability of aPKC to phosphorylate other targets [10].

In contrast to what is known about how Scrib regulates cell polarity, much less is known about how it acts to restrain tissue overgrowth. Studies have suggested that the proliferation and polarity functions of Scrib can be separated [11]. However, whether Scrib operates antagonistically to Crb and aPKC to repress proliferation is not known. In *lgl *mutants, tumour overgrowth can be rescued through reduced levels of aPKC [12], and aPKC over-expression is capable of inducing CycE [13]. However, it is not known if aPKC functions upstream of Lgl, or if Lgl acts to restrain aPKC phosphorylation of alternative key targets that promote epithelial overgrowth. In fact, aPKC can activate Crb through phosphorylation [14] and Crb over-expression in the wing disc promotes epithelial neoplasia similar to loss of function mutants in *scrib*, *dlg *or *lgl *[15]. Thus, deregulated Crb activity could be primarily responsible for neoplastic overgrowth in *scrib *mutants as has been suggested for mutants in the syntaxin *avalanche *(*avl*) [15]. Deciphering the hierarch that operates amongst these key polarity players in *scrib *mutant epithelial neoplasias is required.

Similarly, clarification is needed of how *scrib *mutants cooperate with oncogenes in mediating transformation in *Drosophila*. A number of studies have shown how Ras^ACT ^subverts the pro-apoptotic JNK signalling response in *scrib *mutants into a potent inducer of tumour overgrowth and invasion through the JNK-dependent expression of Matrix metalloproteinase 1 (Mmp1) [16-18]. However, whilst there is agreement on the key role of JNK in mediating cooperative overgrowth, these reports give conflicting conclusions about the role of Scrib. It has been suggested that loss of Scrib contributes JNK-independent roles in promoting cooperation with Ras^ACT ^[17], while others offer evidence that JNK is itself sufficient for the cooperation with Ras^ACT ^[16] and, thus, cell polarity genes such as Scrib repress oncogene-mediated transformation merely by restraining JNK activation. As mammalian studies have recently demonstrated that human Scrib similarly restrains Ras^ACT^-mediated transformation [5], it is important to more fully understand how *Drosophila *Scrib exerts its tumour suppressor function.

In this study, we define for the first time the relationship between Scrib and other cell polarity regulators in the control of cell polarity and proliferation in imaginal discs. Analysing *scrib *mutant clones in the eye disc, we found that although the over-expression of Crb or aPKC mimics many of the *scrib *mutant defects, the excessive proliferation and alterations in cell morphology in *scrib *mutants are independent of Crb but can be rescued through the expression of a dominant negative aPKC transgene. Furthermore, we identified distinct aPKC and JNK-dependent modes by which *scrib *mutants promote oncogene-mediated transformation. Our data support the critical role of JNK signalling in *scrib *mutants in mediating cooperation with Ras^ACT ^and show that JNK is also essential for N^ACT^-driven tumourigenesis. However, our studies also show that aPKC signalling can play a pivotal role in promoting oncogene-mediated tumour overgrowth and these findings are likely to be of relevance to the way in which loss of human Scrib can similarly potentiate oncogene-mediated transformation.

## Methods

### Drosophila stocks

Fly crosses were carried out at 25°C and grown on standard fly media. All clonal analysis was carried out using MARCM (mosaic analysis with repressible cell marker) [19] with *FRT82B *and *eyeless*-*FLP1 *to induce clones and *UAS-mCD8-GFP *to visualize mutant tissue.

The following *Drosophila *stocks were used: *eyFLP1, UAS-mCD8-GFP;;Tub-GAL4 FRT82B Tub-GAL80 *[20]; *msn*^06946 ^[21]; *scrib*^1 ^[22]; *UAS-P35 *[23]; *UAS-bsk*^*DN *^[24]; *crb*^11*A*22 ^[25]; *UAS-crb*^*wt*2*e *^[26]; *UAS-DaPKC*^Δ*N *^[10]; *UAS-DaPKC*^*CAAXWT *^and *UAS-DaPKC*^*CAAXDN *^[14]; *UAS-dRas1*^*V*12 ^[27]; *UAS-N*^*intra *^[28]; *UASp-scrib*^*FL*^19.2 (full length Scrib cDNA cloned into pUASP, this study).

### Immunohistochemistry

Eye/antennal discs and brain lobes were dissected in phosphate-buffered saline (PBS) from wandering third instar larvae and fixed in 4% formaldehyde in PBS. Samples were blocked in either 2% goat serum in PBT (PBS 0.1% Triton X-100) or 5% milk powder/bovine serum albumin in PBS 0.3% Triton X-100. For the detection of S phase cells, a 1 h BrdU (bromodeoxyuridine) pulse was followed by fixation, immuno-detection of green fluorescent protein (GFP), further fixation, acid treatment and immuno-detection of the BrdU epitope. Primary antibodies were incubated with the samples in block overnight at 4°C. Primary antibodies used were: mouse anti-β-galactosidase (Rockland) at 1 in 400, mouse anti-Elav (Developmental Studies Hybridoma Bank) at 1 in 20, rat anti-Cyc E (Helen McNeill) at 1 in 400, rabbit anti-GFP (Invitrogen) at 1 in 1000, mouse anti-BrdU (Becton-Dickinson) at 1 in 50, rabbit anti-Paxillin at 1 in 400 [29]. Secondary antibodies were; anti-mouse/rat/rabbit Alexa647 (Invitrogen) at 1 in 400, anti-mouse/rat biotin (Jackson ImmunoResearch Laboratories) at 1 in 400 and streptavidin-conjugated fluorophores (Jackson ImmunoResearch Laboratories) at 1 in 400. Terminal deoxynucleotidyl transferase mediated X-dUTP nick end labelling (TUNEL) staining was used to detect apoptotic cells (*in situ *cell death detection kit TMR-Red from Roche). F-actin was detected with phalloidin--tetramethylrhodamine isothiocyanate (TRITC; Sigma) at 0.77 μM. Samples were mounted in 80% glycerol.

### Microscopy and image processing

Samples were analysed by confocal microscopy using either Bio-Rad MRC1000 or Olympus FV1000 microscopes. Single optical sections were selected in Confocal Assistant^® ^or Flouroview^® ^software before being processed in Adobe Photoshop^® ^CS2 and assembled into figures in Adobe Illustrator^® ^CS2.

## Results

### JNK signalling is ectopically activated in scrib mutants, but JNK is not responsible for the altered cell morphology or ectopic proliferation in scrib mutant cells

Previously we have shown that *scrib *mutant cells, within clones of tissue in the eye disc, have severely altered cell morphology and exhibit ectopic cell proliferation. However, they do not overgrow because cells die via JNK-mediated apoptosis. Levels of apoptosis were increased in *scrib *mutant mosaic discs and blocking JNK signalling in *scrib *mutant clones by expressing a dominant-negative form of *Drosophila *JNK, Basket dominant negative (Bsk^DN^), dramatically increasing the *scrib *mutant clonal tissue size [2]. In agreement with these observations and other previously published reports [17,30], we confirmed that expressing Bsk^DN ^in *scrib *mutant clones reduced apoptosis in the mutant tissue, although cell death was still observed in some wild type cells abutting the mutant clones (see Additional file 1, panels A-C). Furthermore, using a reporter of JNK signalling, the *lacZ *enhancer trap, *misshapen *(*msn*)-*lacZ *[31], we also confirmed that JNK signalling was ectopically activated within some *scrib *mutant cells, including those undergoing apoptosis, and that expressing Bsk^DN ^in *scrib *mutant clones effectively prevented the ectopic expression of *msn-lacZ *in the mutant tissue (see Additional File 1, panels D-G).

**Figure 1:**
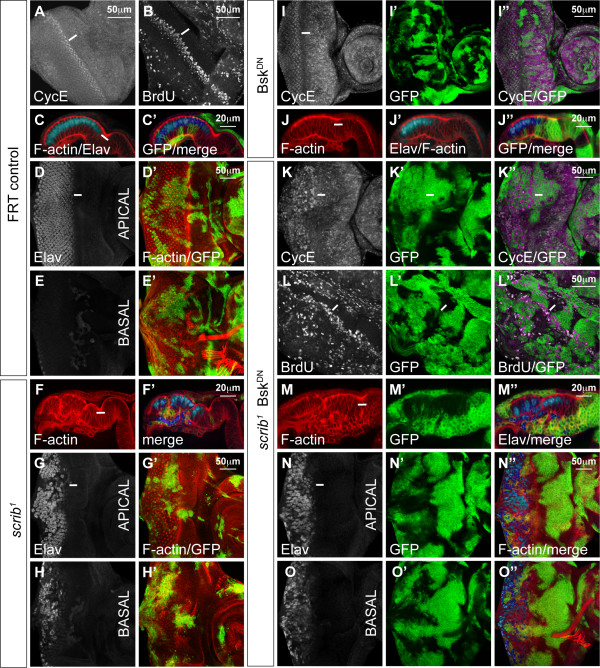
***scrib *mutant clones expressing Bsk^DN ^ectopically proliferate and have disrupted cell morphology**. Third instar larval eye/antennal imaginal discs (posterior to the left in all figures) containing *eyFLP*-induced MARCM clones expressing mCD8-GFP (green) to mark mutant tissue. Planar optical sections are shown (apical and basal sections through the same disc for some samples), except C, F, J, M which are cross sections (apical up). Grey scale is CycE (A, I, K), BrdU (B, L) and Elav (D-H, J, M-O). Red is phalloidin to mark F-actin (C-H, J, M-O). A white bar indicates the location of the MF. (A-E) *FRT82B*. Control eye disc clones exhibit the normal pattern of CycE expression (A) and BrdU incorporation (B) with asynchronous cycles anterior to the MF, a synchronous band of S phases just posterior to the MF and a further round of division of unspecified cells in the more posterior portion of the eye disc. In cross section (C), the columnar epithelial cell morphology is apparent, with apically localized photoreceptor cell nuclei (Elav positive), which are only seen in apical planar sections (D) and not in more basal sections (E). (F-H) *FRT82B scrib*^1^. *scrib *mutant cells have altered cell morphology with many cells contracting beneath the epithelium resulting in the aberrant localization of Elav-positive photoreceptor nuclei basally within the eye disc. (I-J) *FRT82B UAS-bsk*^*DN*^. Bsk^DN^-expressing clones exhibit a normal pattern of CycE expression (I), and, in cross section, normal cell morphology (J). (K-O) *FRT82B scrib*^1 ^*UAS-bsk*^*DN*^. Expressing Bsk^DN ^in *scrib *mutant clones increases clonal tissue size and mutant cells ectopically express CycE (K) and ectopically incorporate BrdU (L) posterior to the MF, although they arrest proliferation normally within the MF, and have aberrant cell morphology with many photoreceptor nuclei localized basally within the epithelium (M-O).

Thus, having confirmed that ectopic JNK signalling in *scrib *mutant cells promoted cell death, we next wished to determine if any of the other *scrib *mutant defects, including the ectopic cell proliferation and altered cell morphology, were also dependent upon JNK. Proliferation in the eye disc follows a stereotypical pattern that can be visualized by CycE levels and bromedeoxyuridine (BrdU) incorporation. Cells normally arrest cell proliferation within the morphogenetic furrow (MF) and undergo a synchronous S phase just posterior to the MF before commencing differentiation, although some unspecified cells undergo a further round of division more posteriorly (Figure 1A, B). Differentiation in the posterior half of the eye disc can be marked with Elav staining to identify the apically localized nuclei of the developing photoreceptor cells (Figure 1C-E), although in *scrib *mutant clones the disruption to cell morphology results in photoreceptor nuclei being aberrantly localized basally within the epithelium (Figure 1F-H). Blocking JNK signalling by expressing Bsk^DN ^did not alter the normal pattern of cell proliferation or morphology within the eye disc (Figure 1I, J), however, *scrib *mutant cells expressing Bsk^DN ^showed ectopic cell proliferation posterior to the MF (Figure 1K, L) and aberrant cell morphology similar to *scrib *mutants alone (Figure 1M). The mutant tissue tended to drop beneath the epithelium resulting in photoreceptor cell nuclei of both mutant and wild type cells being aberrantly localized basally within the epithelium (Figure 1N, O). It was therefore apparent that, while *scrib *mutant cells were eliminated by JNK-dependent apoptosis, the proliferative and cell morphology defects of *scrib *mutants were JNK-independent.

### The scrib mutant phenotype is phenocopied by Crb over-expression, but is not dependent upon Crb

Analysis in the embryo has established that cell polarity is regulated through antagonistic interactions between Scrib/Dlg/Lgl and two different polarity complexes, the Crumbs complex (including Crb, Sdt and Patj) and the Baz complex (including Baz, aPKC and Par6). To determine if this hierarchal relationship is also operative in the eye disc, we began by examining the effects of Crb loss-of-function and Crb over-expression in the eye disc.

Loss-of-function *crb *clones, using the null allele *crb*^11*A*22 ^[25], exhibited no apparent defects in differentiation or cell morphology (see Additional file 2, panels A-B), although during pupal development cell morphology defects become apparent within the developing photoreceptor cells [32,33]. In contrast, third instar larval eye disc clones over-expressing a wild-type Crb transgene were small and mutant cells tended to be excluded from the epithelium with severely altered, more rounded, cell morphology. If, however, JNK signalling was blocked within the Crb-expressing tissue by co-expressing Bsk^DN^, clones became considerably larger and also exhibit ectopic cell proliferation posterior to the MF (see Additional file 2, panels C-F). Similar overgrowth and polarity defects, but not JNK-dependent cell death, have been described when Crb was over-expressed in the wing disc epithelium [15].

**Figure 2:**
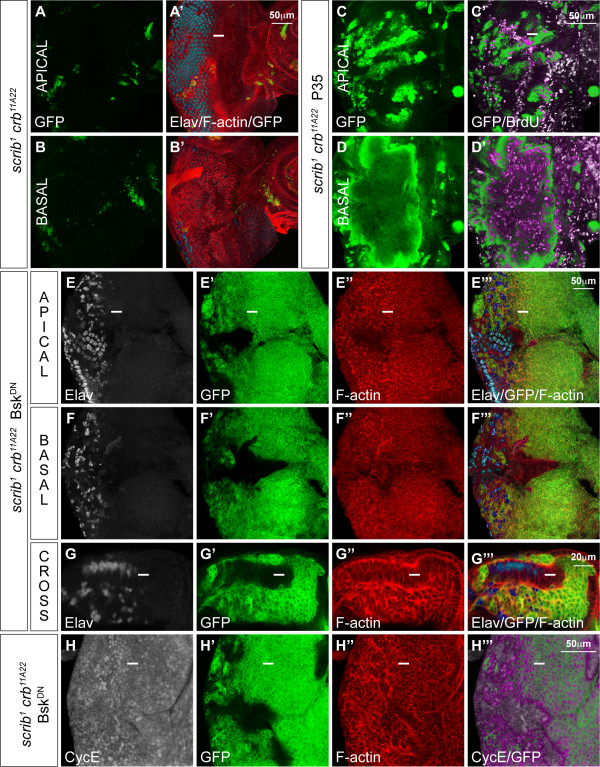
***scrib crb *double mutant cells show similar defects to *scrib *mutant cells**. *eyFLP*-induced MARCM clones (green) shown in planar and cross section. Grey scale is Elav (A, B, E-G), BrdU (C, D) and CycE (H). Phalloidin marks F-actin in red (A, B, E-H). A white bar indicates the location of the MF. (A, B) *FRT82B crb*^11*A*22 ^*scrib*^1^. *scrib crb *double mutant clones are small, and under-represented relative to surrounding non-clonal tissue in both apical and basal sections of the eye/antennal disc. (C, D) *UAS-P35*; *FRT82B crb*^11*A*22 ^*scrib*^1^. *scrib crb *double mutant clones expressing the caspase inhibitor P35 are considerably larger than (A), with most mutant tissue being extruded basally and showing ectopic proliferation. (E-H) *FRT82B crb*^11*A*22 ^*scrib*^1 ^*UAS-bsk*^*DN*^. The expression of Bsk^DN ^in *scrib crb *double mutant clones results in large clones with altered cell morphology and many Elav positive nuclei in mutant and adjacent wild type tissue being mislocalized basally within the epithelium (E-G). The mutant cells ectopically express CycE posterior to the MF (H).

The similarity in phenotypes between *scrib *mutants and Crb over-expression raised the possibility that ectopic Crb activity could account for the defects in *scrib *mutant cells. To test this we generated *scrib*^1 ^*crb*^11*A*22 ^double mutant clones. Like *scrib *mutant cells, *scrib crb *double mutant cells had altered cell morphology and were under-represented in mosaic eye discs (Figure 2A, B). If cell death was prevented through the expression of the caspase inhibitor P35, clone viability was enhanced. The mutant cells showed extreme alterations in cell morphology and most mutant tissue no longer formed a columnar epithelium, but it was contracted and basally extruded beneath the epithelium where it continued to proliferate ectopically (Figure 2C, D). Furthermore, if JNK signalling was blocked in *scrib crb *double mutant clones, not only did clones massively overgrow, taking over most of the eye disc, but, like *scrib *mutants, cell morphology remained perturbed (Figure 2E-G) and mutant cells continued to ectopically express CycE posterior to the MF (Figure 2H). These data indicate that while Crb over-expression reproduces many of the *scrib *mutant defects, ectopic Crb activity is not responsible for the *scrib *mutant phenotype and, therefore, Crb is likely to function either upstream or independently from Scrib in the larval eye disc.

### aPKC signalling is required for the polarity and proliferation defects in scrib mutant cells

aPKC is a component of the Baz complex and can function in opposition to Scrib/Dlg/Lgl. Previously it has been shown that ectopic expression of aPKC in *Drosophila *can disrupt epithelial cell morphology and induce CycE expression [13], although this was not in a clonal context. Therefore, in order to verify that the over-expression of aPKC could mimic the *scrib *mutant phenotype in the eye, we over-expressed wild-type aPKC incorporating a membrane-tethering CAAX motif (aPKC^CAAXWT^) in eye disc clones [14]. This produced a variable phenotype but generally led to only mild defects in tissue organization and very weak ectopic CycE expression (data not shown). In order to investigate the more extreme consequences of aPKC activation we analysed clones of eye disc tissue ectopically expressing an activated version of aPKC lacking its N-terminal regulatory domain (aPKC^ΔN^) [10]. This resulted in small eye disc clones, however, blocking JNK signalling in the aPKC^ΔN^-expressing clones restored clone viability and most of the mutant tissue had aberrant morphology and was extruded basally to form large masses of undifferentiated tissue that ectopically proliferated posterior to the MF (see Additional file 3). Thus, like Crb over-expression, the over-expression of aPKC^ΔN ^reproduced many of the *scrib *mutant defects, including the alterations in cell morphology, ectopic cell proliferation and JNK-dependent cell death.

**Figure 3:**
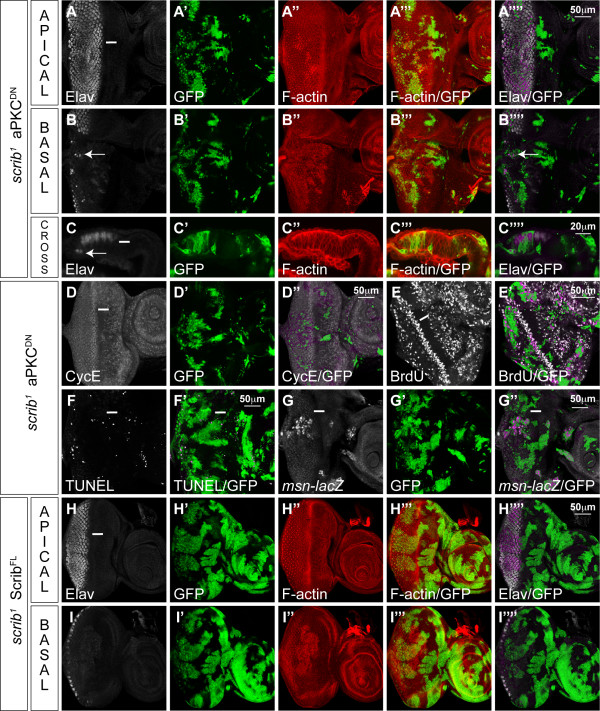
**aPKC^CAAXDN ^rescues *scrib *mutant morphology and proliferation defects, but does not prevent JNK-mediated apoptosis**. *eyFLP*-induced MARCM clones (green). Grey scale is Elav (A-C, H, I), CycE (D), BrdU (E), TUNEL (F) and β-Gal to detect *msn*^06946^-*lacZ *enhancer trap activity (G). Phalloidin marks F-actin in red (A-C, H, I). A white bar indicates the location of the MF. (A-G) *FRT82B scrib*^1 ^*UAS-DaPKC*^*CAAXDN*^. Expression of aPKC^CAAXDN ^in *scrib *mutant clones rescues most cell morphology defects and normalizes the regular pattern of differentiation in the eye disc (A-C) with only occasional photoreceptor nuclei dropping basally at the edges of some mutant clones (arrow in B and C). The mutant cells no longer ectopically express CycE (D) or ectopically proliferate posterior to the MF (E), but the mutant cells still die as seen with TUNEL detection (F) and JNK signalling is still ectopically activated in some mutant tissue, as measured by the activity of the *msn-lacZ *enhancer trap (G). (H, I) *UAS-scribble*^*FL*^19.2; *FRT82B scrib*^1^. Expression of a full-length Scrib transgene in *scrib *mutant clones completely rescues the mutant cell morphology defects as well as clonal tissue size throughout the eye/antennal disc.

To determine if the *scrib *mutant defects could be due to deregulated aPKC activity we utilized a transgene expressing a kinase-dead, CAAX membrane-tethered, allele of aPKC (aPKC^CAAXDN^) [14]. The expression of aPKC^CAAXDN ^in otherwise wild-type clones of tissue produced no discernible defects in cell morphology, proliferation or differentiation during larval stages of development (data not shown). Strikingly, however, the expression of aPKC^CAAXDN ^in *scrib *mutant clones restored normal cell morphology to the mutant tissue posterior to the MF. Elav and phalloidin staining generally revealed a normal regular array of differentiating ommatidial clusters in *scrib *mutant clones expressing aPKC^CAAXDN^, although sometimes clonal borders showed separation between the mutant and wild-type tissue resulting in tissue scars (data not shown) and occasional basally retracted mutant photoreceptor nuclei (Figure 3A-C). Furthermore, *scrib *mutant clones expressing aPKC^CAAXDN ^no longer exhibited ectopic CycE or BrdU incorporation posterior to the MF (Figure 3D, E), although ectopic CycE and BrdU positive cells were still sometimes observed surrounding the mutant clones of tissue (data not shown). Such a phenomena is reminiscent of the non-cell autonomous compensatory cell proliferation that can be induced by dying cells within imaginal discs [reviewed in [34]]. Indeed, although the expression of aPKC^CAAXDN ^in *scrib *mutant clones rescued most of the *scrib *mutant defects, the viability of the mutant tissue remained poor and the remnants of many apoptotic cells were evident. TUNEL detection confirmed that there were dying cells in *scrib *mutant clones expressing aPKC^CAAXDN ^(Figure 3F) and the ectopic expression of the JNK pathway reporter, *msn-lacZ*, in the mutant tissue suggested that this was due to a failure to rescue JNK-dependent cell death (Figure 3G). The failure of aPKC^CAAXDN ^to rescue JNK-dependent cell death was not simply due to an inherent inability to fully rescue cell survival in *scrib *mutant clones caused by a delay in transgene expression, since a full length Scrib transgene fully restored cell morphology and normal clone size to *scrib *mutant cells throughout the eye/antennal disc (Figure 3H, I). Thus, while aPKC^CAAXDN ^rescues the cell morphology and proliferative defects of *scrib *mutant clones, it is not capable of blocking JNK activation in the mutant tissue.

### Blocking both aPKC and JNK signalling rescues scrib mutant morphology, proliferation and viability defects

**Figure 4:**
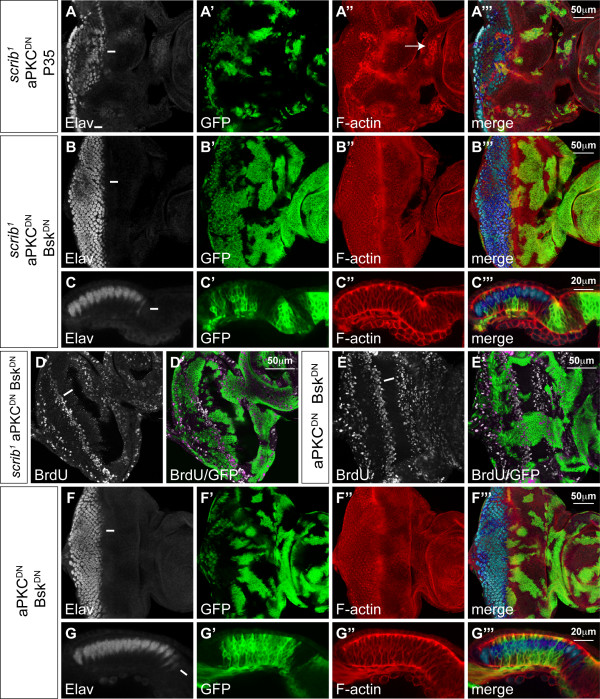
**Expressing Bsk^DN ^and aPKC^CAAXDN ^in *scrib *mutant clones fully rescues the mutant phenotype**. *eyFLP*-induced MARCM clones (green). Grey scale is Elav (A-C, F, G) and BrdU (D, E). Phalloidin marks F-actin in red (A-C, F, G). A white bar indicates the location of the MF. (A) *FRT82B scrib*^1 ^*UAS-DaPKC*^*CAAXDN *^*UAS-P35*. Co-expression of P35 with aPKC^CAAXDN ^in *scrib *mutant clones does not dramatically increase mutant tissue viability and results in non-cell autonomous tissue folding, and rounded cell morphology in some mutant cells (arrow). (B-D) *FRT82B scrib*^1 ^*UAS-DaPKC*^*CAAXDN *^*UAS-bsk*^*DN*^. Co-expression of Bsk^DN ^with aPKC^CAAXDN ^in *scrib *mutant clones rescues the mutant cell morphology and viability defects in both eye and antennal disc regions (B, C) and restores the normal pattern of cell proliferation posterior to the MF (D). (E-G) *FRT82B UAS-DaPKC*^*CAAXDN *^*UAS-bsk*^*DN*^. Co-expression of Bsk^DN ^with aPKC^CAAXDN ^in clones has no discernible effect on cell proliferation (E) or cell morphology and differentiation (F, G).

In an attempt to rescue the cell death phenotype of *scrib *mutants expressing aPKC^CAAXDN^, we co-expressed the apoptosis inhibitor P35 in the mutant clones. This, however, failed to significantly rescue clone size and only served to enhance the mutant phenotype. Non-cell autonomous tissue folding that distorted the shape of the disc was apparent and, in some clones, cells adopted a more rounded morphology (Figure 4A). As the expression of P35 was not capable of blocking JNK activation in *scrib *mutants (data not shown), the data suggest that blocking cell death in *scrib *mutants is not enough to fully rescue the mutant phenotype if JNK remains active.

In contrast to the effects of P35, if JNK signalling was blocked in *scrib *mutant cells expressing aPKC^CAAXDN^, by co-expressing Bsk^DN^, not only was cell viability dramatically restored but the mutant tissue also exhibited normal morphology (Figure 4B, C), although occasional scaring and basally located photoreceptor cell nuclei were still sometimes observed at the edges of mutant clones (data not shown). Furthermore, BrdU incorporation confirmed that the normal pattern of cell proliferation was restored to the mutant tissue (Figure 4D). Otherwise wild-type clones of tissue co-expressing aPKC^CAAXDN ^and Bsk^DN ^showed a normal pattern of cell proliferation and morphology (Figure 4E-G). Thus, virtual complete suppression of the *scrib *mutant phenotype could be achieved by blocking both aPKC and JNK signalling.

In summary, distinct aPKC and JNK-dependent defects can be genetically separated in *scrib *mutants. Blocking aPKC activity in *scrib *mutant clones restores most of the mutant defects, including the alterations in cell morphology and the ectopic cell proliferation, but it does not rescue the mutant cells from JNK-mediated cell death. Blocking aPKC and JNK signalling together restores mutant clone viability and results in almost complete suppression of the mutant phenotype.

### JNK, but not aPKC, signalling is necessary for Ras^ACT^-driven tumour overgrowth of scrib mutants

**Figure 5:**
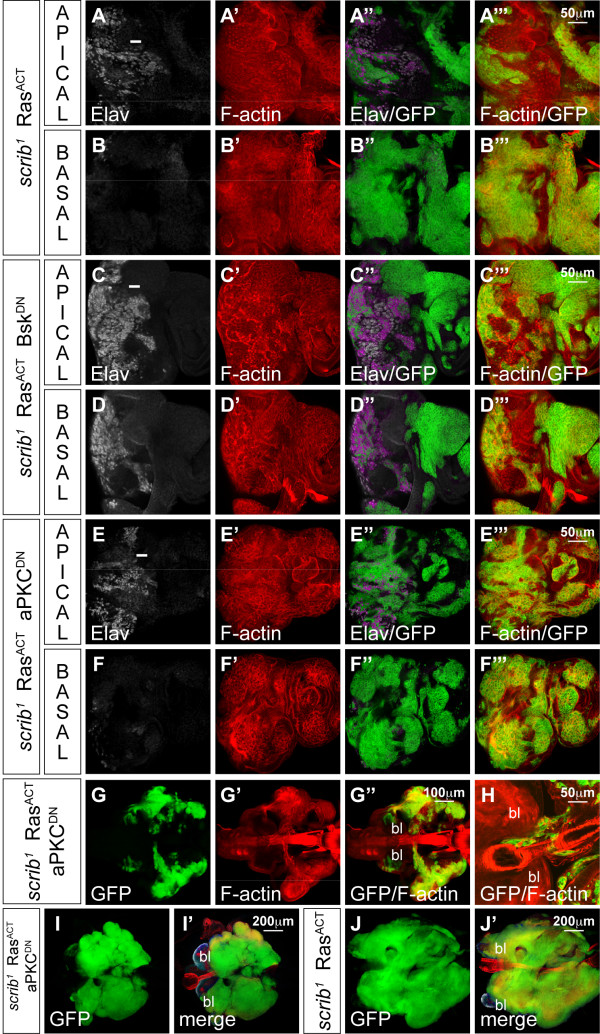
**Expression of JNK^DN^, but not aPKC^CAAXDN^, in *scrib*^1 ^+ Ras^ACT ^tumours restores differentiation**. Larval eye/antennal imaginal discs, with brain lobes (bl) attached (G-J), containing *eyFLP*-induced MARCM clones (green) at day 5 (A-F), day 7 (G, H) and day 9 (I, J). Grey scale is Elav and Red is phalloidin to mark F-actin. A white bar indicates the location of the MF. (A, B) *UAS-dRas1*^*V*12^; *FRT82B scrib*^1^. Expression of Ras^ACT ^in *scrib *mutant clones results in tumour overgrowth basally. In apical sections, some differentiation is still observed in mutant tissue, although more basal sections show tumour cells overgrowing without differentiation. (C, D) *UAS-dRas1*^*V*12^; *FRT82B scrib*^1 ^*UAS-bsk*^*DN*^. Co-expression of Bsk^DN ^with Ras^ACT ^in *scrib *mutant clones restores differentiation to the tumour cells in both apical and basal sections. (E-I) *UAS-dRas1*^*V*12^; *FRT82B scrib*^1 ^*UAS-DaPKC*^*CAAXDN*^. Co-expression of aPKC^CAAXDN ^with Ras^ACT ^in *scrib *mutant clones fails to restore differentiation to the tumour cells (E, F) that continue to massively overgrow and invade between the brain lobes (G, H), resulting in neoplasias at day 9 (I) that are only marginally smaller than day 9 *scrib*^1 ^+ Ras^ACT ^tumours (J).

In addition to the proliferation and cell death defects of *scrib *mutant clones, we have also observed that they cooperate with activated alleles of *dRas1 *(Ras^ACT^) Ras^ACT ^or *Notch *(N^ACT^) to repress pupal development and, throughout an extended 'giant larvae' phase of development, form massive and invasive tumours [2,3]. *scrib*^- ^+ Ras^ACT ^tumour cells grow out basally from the eye disc, fail to differentiate (Figure 5A, B) and appear to invade the brain lobes along F-actin rich cables extending from between the eye/antennal disc to the brain, eventually leading to a fusion between the eye discs, brain lobes and surrounding tissues (see Additional file 4, panels A-D). Like the proliferative and cell morphology defects of *scrib *mutants, the cooperation with Ras^ACT ^was independent of Crb function, since the expression of Ras^ACT ^in *scrib*^1 ^*crb*^11*A*22 ^double mutant clones also resulted in the formation of large neoplasias (see Additional file 4, panel E). Therefore, using the distinct JNK and aPKC-dependent phenotypes of *scrib *mutants that we had defined, we were then interested in investigating the contribution of each of these to the Ras-driven tumourigenic phenotype.

Consistent with previous reports [16,17], we found that blocking JNK signalling in *scrib*^- ^+ Ras^ACT ^tumours, by co-expressing Bsk^DN^, restored pupation to the tumour-bearing larvae and repressed tumour invasion (see Additional file 4, panels F, G). Proteins involved in both cell migration and invasion, including the matrix metalloproteinase, Mmp1 (data not shown) [16,18] and the integrin-associated scaffolding protein, Paxillin (Pax), were up-regulated in *scrib *mutant clones and at the invasive front of *scrib*^- ^+ Ras^ACT ^tumours, in a JNK-dependent manner (see Additional file 5). Furthermore, the JNK reporter, *msn-lacZ*, was strongly activated in tumour cells located between the brain lobes, thus correlating JNK activity with tumour cell invasion (see Additional file 6). However, blocking JNK signalling within *scrib*^- ^+ Ras^ACT ^tumours not only prevented tumour cell invasion, it also abrogated tumour overgrowth throughout the extended larval stage of development. Indeed, examination of differentiation in the eye disc revealed that while *scrib*^- ^+ Ras^ACT ^tumours grew basally within the eye disc and failed to express Elav, blocking JNK signalling restored the ability of the tumour cells to differentiate (Figure 5C, D).

JNK signalling in *scrib *mutants is therefore required for both invasion and loss of differentiation during Ras^ACT^-mediated transformation but does loss of Scrib also contribute aPKC-dependent activities that promote Ras^ACT^-driven tumourigenesis? To address this issue, we co-expressed aPKC^CAAXDN ^with Ras^ACT ^in *scrib *mutant clones. Although aPKC^CAAXDN ^was capable of rescuing *scrib *mutant defects in cell morphology and proliferation (see above), it was unable to repress Ras^ACT^-induced tumour development. Examination of differentiation by Elav staining confirmed that *scrib*^- ^+ Ras^ACT ^+ aPKC^CAAXDN ^tumour tissue remained undifferentiated in the basal sections of the eye disc (Figure 5E, F). Furthermore, the tumour-bearing larvae failed to pupate and the tumours continued to overgrow and invade the adjacent brain lobes throughout a 'giant larvae' phase of development (Figure 5G, H), resulting in massive and fused tumour masses, only marginally smaller than *scrib*^- ^+ Ras^ACT ^controls (Figure 5I, J). Thus, as aPKC^CAAXDN ^is capable of rescuing most of the *scrib *mutant defects apart from JNK-mediated cell death, the failure of aPKC^CAAXDN ^to block *scrib*^- ^+ Ras^ACT ^tumourigenesis supports the hypothesis that JNK signalling alone is both necessary and sufficient in cooperation with Ras^ACT ^to lead to neoplastic transformation [16].

### JNK signalling is necessary, and aPKC signalling potentiates, scrib mutant cooperation with N^ACT^

**Figure 6:**
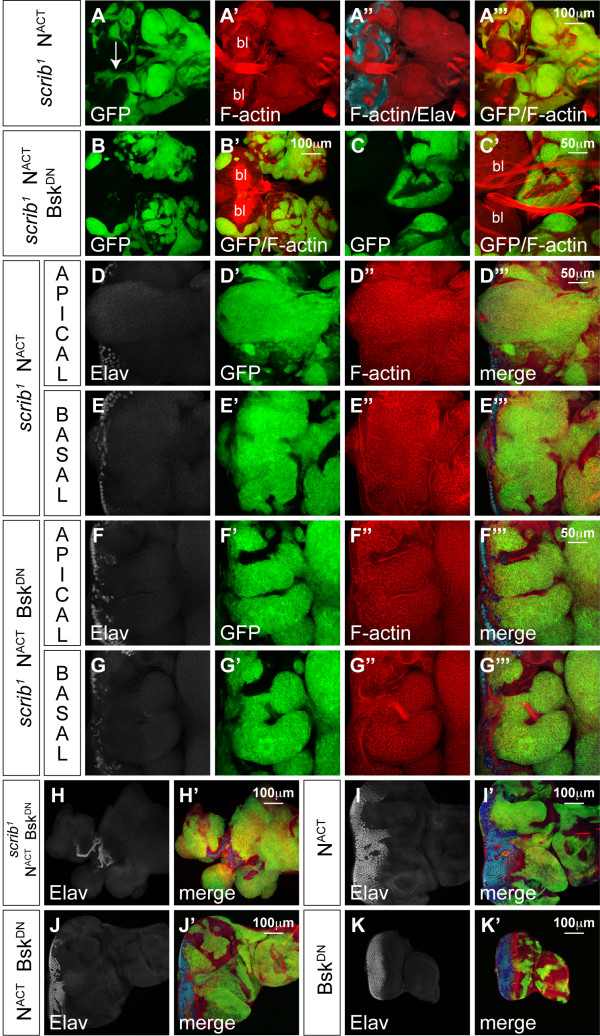
**Bsk^DN ^blocks *scrib*^1 ^+ N^ACT ^neoplastic overgrowth but does not restore differentiation**. Larval eye/antennal imaginal discs containing *eyFLP*-induced MARCM clones (green) at approximately day 7 (A) and day 5 (B-K). Eye discs remain attached to each brain lobe (bl) in A-C. Grey scale is Elav and Red is Phalloidin to mark F-actin. (A, D, E) *UAS-N*^*intra*^; *FRT82B scrib*^1^. Expression of N^ACT ^in *scrib *mutant clones results in tumour overgrowth with cells appearing to migrate (arrow) between the brain lobes (Elav positive) at day 7 (A) and failing to differentiate in apical and basal sections of the eye disc (D, E). (B, C, F-H) *UAS-N*^*intra*^; *FRT82B scrib*^1 ^*UAS-bsk*^*DN*^. Co-expression of Bsk^DN ^with N^ACT ^in *scrib *mutant clones results in larvae pupating at day5/6, thus precluding analysis of invasion at day 7. However, at day 5 no invasion is seen to occur between the brain lobes (B, C), despite tumour cells remaining undifferentiated (F, G) and forming large benign overgrowths (H). (I) *UAS-N*^*intra*^; *FRT82B*. Expression of N^ACT ^alone in clones results in massively overgrown eye antennal discs. (J) *UAS-N*^*intra*^; *FRT82B UAS-bsk*^*DN*^. Co-expression of N^ACT ^with Bsk^DN ^also results in massively overgrown eye/antennal discs. (K) *FRT82B UAS-bsk*^*DN*^. Bsk^DN^-expressing eye/antennal discs are of normal size and differentiation.

As *scrib *mutants also cooperate with N^ACT ^to produce non-differentiated tumours that invade and fuse with the brain lobes (Figure 6A), we also investigated whether JNK was essential for N-driven tumourigenesis. Indeed, like *scrib*^- ^+ Ras^ACT ^tumours, expressing Bsk^DN ^in *scrib*^- ^+ N^ACT ^tumours rescued the extended larval development and 'giant larvae' phenotype characteristic of unrestrained neoplastic overgrowth and repressed tumour invasion (Figure 6B, C). However, in contrast to Ras^ACT^-driven tumours, blocking JNK signalling in *scrib*^- ^+ N^ACT ^tumours failed to restore differentiation (Figure 6D-G) and the eye antennal discs formed massive, and often amorphous masses, of benign tissue overgrowth prior to the larvae pupating at day5/6 (Figure 6H). The benign tumour overgrowth was largely N-dependent, since expressing N^ACT ^alone (Figure 6I), or N^ACT ^with Bsk^DN ^(Figure 6J), in eye disc clones also blocked differentiation and resulted in massively overgrown eye/antennal discs, albeit without the amorphous structure characteristic of the loss of cell polarity in the *scrib*^- ^+ N^ACT ^+ Bsk^DN ^clones. In contrast, Bsk^DN^-expressing mosaic discs were of normal size and differentiation (Figure 6K).

**Figure 7:**
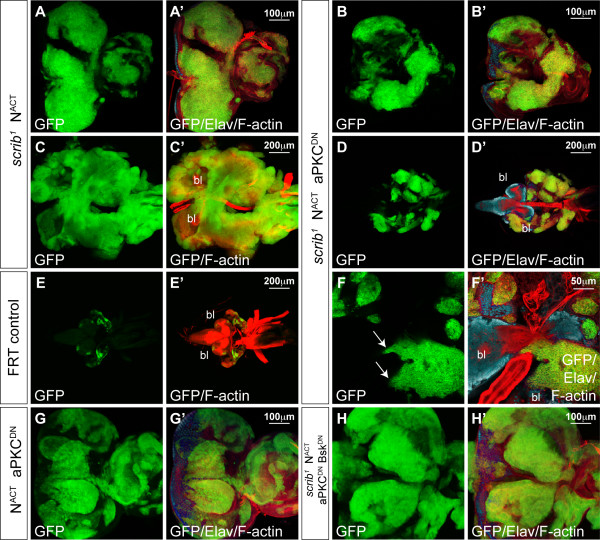
**aPKC^CAAXDN ^restrains *scrib*^1^+ N^ACT ^neoplastic overgrowth**. Larval eye/antennal imaginal discs containing *eyFLP*-induced MARCM clones (green) at day 5 (A, B, E, G-H) and day 9 (C, D, F). Grey scale is Elav and Red is Phalloidin to mark F-actin. (A, C) *UAS-N*^*intra *^*FRT82B scrib*^1^. Expression of N^ACT ^in *scrib *mutant clones results in large tumours at day 5 (A) and these become massive (compare to *FRT82B *control clones in E) and fuse with the brain lobes (bl) by day 9 (C). (B, D, F) *UAS-N*^*intra*^; *FRT82B scrib*^1 ^*UAS-DaPKC*^*CAAXDN*^. Co-expression of aPKC^CAAXDN ^with N^ACT ^in *scrib *mutant clones fails to restore tumour differentiation but retards tumour overgrowth at day 5 (B compared to A), and this becomes more apparent by day 9 (D compared to C), although tumour cells are still observed between the brain lobes (arrows in F). (G) *UAS-N*^*intra*^; *FRT82B UAS-DaPKC*^*CAAXDN*^. Co-expression aPKC^CAAXDN ^with N^ACT ^does not abrogate N^ACT^-driven overgrowth of the eye/antennal disc. (H) *UAS-N*^*intra*^; *FRT82B scrib*^1 ^*UAS-bsk*^*DN *^*UAS-DaPKC*^*CAAXDN*^. Expression of aPKC^CAAXDN ^and Bsk^DN ^with N^ACT ^in *scrib *mutant clones prevents neoplastic tumor overgrowth and restores the characteristically overgrown mosaic discs of N^ACT^-expressing clones.

To determine whether the loss of cell polarity and proliferative defects of *scrib *mutants contributed to N^ACT^-driven tumourigeneis, we again made use of the observation that aPKC^CAAXDN ^rescues most of the *scrib *mutant defects but does not stop JNK-mediated cell death. Expressing aPKC^CAAXDN ^in *scrib*^- ^+ N^ACT ^tumours did not prevent neoplastic overgrowth and many larvae failed to pupate and entered an extended 'giant larvae' phase of the development, consistent with JNK signalling being sufficient for cooperation with N^ACT^, as it is for Ras^ACT^. However, tumour overgrowth was strikingly restrained compared to *scrib*^- ^+ N^ACT ^tumours. By day 5, *scrib*^- ^+ N^ACT ^+ aPKC^CAAXDN ^tumour size was only mildly reduced compared to that of the controls (Figure 7A, B). However, by day 9, although the tumour continued to grow, it was significantly smaller than the massive *scrib*^- ^+ N^ACT ^overgrowths (Figure 7C, D). Despite this reduction in tumour growth, *scrib*^- ^+ N^ACT ^+ aPKC^CAAXDN ^neoplasias still invaded and fused with the adjacent brain lobes (Figure 7F). Thus, although blocking aPKC function was not enough to prevent neoplasia, aPKC signalling was required to enhance the *scrib*^- ^+ N^ACT ^tumour overgrowth.

The tumour growth promoting role of aPKC in *scrib*^- ^+ N^ACT ^neoplasias could have reflected a direct requirement for aPKC signalling in N^ACT^-driven hyperplasia. However, expressing aPKC^CAAXDN ^with N^ACT ^in otherwise wild-type eye disc clones resulted in overgrown mosaic discs (Figure 7G) similar to N^ACT ^(Figure 6I). This suggested that aPKC signalling was only required to promote N^ACT^-dependent hyperplasia when Scrib function was lost. Furthermore, as JNK is activated in *scrib*^- ^+ aPKC^CAAXDN ^clones, it seemed likely that JNK signalling was responsible for restraining *scrib*^- ^+ N^ACT ^+ aPKC^CAAXDN ^tumour overgrowth. Indeed, blocking JNK signalling in *scrib*^- ^+ N^ACT ^+ aPKC^CAAXDN ^clones blocked tumour formation, consistent with the key requirement for JNK in promoting neoplastic overgrowth, and restored the characteristically overgrown mosaic disc phenotype of N^ACT^-expressing clones (Figure 7H). Thus, blocking both JNK and aPKC signalling completely suppressed the ability of *scrib *mutants to cooperate with oncogenic N signalling and overcame the aPKC^CAAXDN^-dependent restraint in *scrib*^- ^+ N^ACT ^tissue overgrowth.

Therefore, in summary, JNK signalling exerts opposing tumour-promoting and tumour-repressing forces upon N^ACT^-driven neoplasia. While JNK is critically required for neoplastic overgrowth in cooperation with N^ACT^, as it is for Ras^ACT^, JNK can also restrain N-driven overgrowth and the loss of Scrib can help overcome the JNK-dependent restraint through aPKC-dependent pathways.

## Discussion

**Figure 8:**
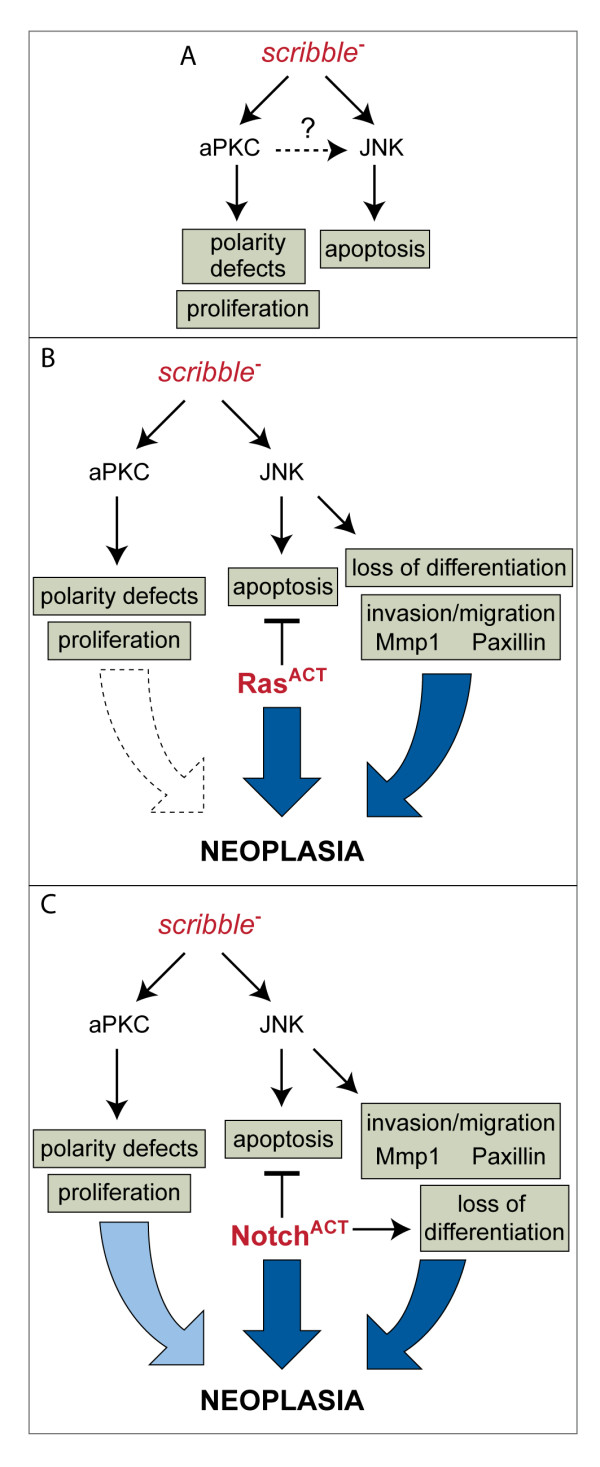
**Model depicting the pathways through which *scrib *mutants promote tumorigenesis**. (A) In *scrib *mutant cells, inappropriate aPKC activity leads to alterations in cell polarity/morphology and excessive cell proliferation that is restrained through JNK-dependent apoptosis. Although distinct aPKC and JNK-dependent pathways could be genetically separated in *scrib *mutants, it is possible that aPKC-dependent defects, refractory to aPKC^CAAXDN^-mediated inhibition, still drive JNK activation. (B) Expressing Ras^ACT ^in *scrib *mutant cells blocks JNK-mediated apoptosis and unveils a role for JNK in promoting loss of differentiation, tumour overgrowth and invasion. aPKC signalling exerts only a minor role in promoting tumour overgrowth. (C) Expressing N^ACT ^in *scrib *mutant cells blocks differentiation and promotes JNK-mediated tumour overgrowth and invasion. aPKC signalling promotes tumour overgrowth through either increased cell proliferation or cell survival to counteract a JNK-dependent restraint on tumour overgrowth.

In this study we have extended our original analysis of *scrib *mutant phenotypes in the eye disc epithelium to investigate the relationship between *scrib *and other cell polarity regulators in the control of epithelial neoplasia (Figure 8). This has revealed that the hierarchical relationship between Scrib, Crb and aPKC that regulates epithelial cell polarity in the embryo also controls neoplastic overgrowth in the eye disc, with aPKC being the likely effector of the cell polarity and proliferation defects in *scrib *mutants. We have also identified distinct JNK and aPKC-dependent modes by which *scrib *mutants cooperate with oncogenes in tumourigenic overgrowth and this has the potential to impact upon our understanding of how loss of human Scrib can also promote oncogene-mediated transformation.

### The relationship between Scrib, Crb and aPKC

Our genetic analysis in eye disc clones indicates that, although Crb over-expression reproduces many of the *scrib *mutant defects, the *scrib *phenotype is not dependent upon Crb activity. This supports the epistatic relationship between *scrib *and *crb *described in the embryo, with the *scrib *mutant phenotype being dominant over the *crb *mutant phenotype, and suggesting that Crb acts upstream or independently of Scrib [8]. In contrast, the strong rescue of *scrib *mutant defects by expressing a dominant negative aPKC transgene suggests that aPKC either acts to inactivate Scrib and blocking aPKC restores Scrib activity as has been proposed for Lgl, or deregulated aPKC activity accounts for the *scrib *mutant phenotype. We favour the latter possibility because of the inability of aPKC^CAAXDN ^to rescue JNK-mediated cell death of *scrib *mutant tissue which is more consistent with aPKC functioning downstream of *scrib*. However, complex cross talk between the polarity regulators is likely to exist. Crb over-expression phenotypes can also be suppressed by aPKC^CAAXDN ^co-expression, and aPKC can phosphorylate Crb to modulate its activity [14]. Similarly, aPKC can phosphorylate and inactivate Lgl, although Lgl also functions genetically upstream of aPKC in restraining the formation of neuroblastomas, by acting as a competitive substrate of aPKC and impeding aPKC's ability to phosphorylate and inactivate Numb [35]. In mammals, Scrib can also function upstream of aPKC via the correct localization of Cdc42 during cell migration [36,37]. However, in this context loss of Scrib appears to impair localized aPKC activity, suggesting that the relationship between Scrib and aPKC activity can vary in different contexts.

If Scrib does function upstream of aPKC in *Drosophila*, then either loss of Scrib promotes JNK activation independently of aPKC, or, alternatively, deregulated aPKC activity in *scrib *mutants can induce JNK-mediated cell death through a mechanism that is refractory to aPKC^CAAXDN^(kinase dead)-mediated inhibition. Our own work indicates that ectopic aPKC expression can induce JNK-dependent cell death, although whether the kinase dead form of aPKC can block this death is not known. In any event, the relationship between *scrib *and JNK is not likely to be direct since JNK was not activated in all *scrib *mutant tissue and was often associated with clonal borders. This is more consistent with JNK being indirectly activated due to either alterations in cell adhesion or signalling. Consistent with this, *scrib *mutant clones expressing both aPKC^CAAXDN ^and Bsk^DN ^still showed occasional scaring at clonal edges suggestive of an impaired cell adhesion.

A relatively small number of neoplastic tumour suppressor mutants have been described in *Drosophila *and, apart from the junctional/scaffold tumour suppressors of *scrib*, *dlg *and *lgl*, the other group of genes, *Rab5*, *avl*, *erupted *and *vps25*, regulate endocytic pathways. Interestingly, *avl *mutant hyperplasia is also rescued by the expression of aPKC^CAAXDN ^and this was suggested to reflect the ability of aPKC^CAAXDN ^to reduce Crb activity, since Crb levels were elevated and mislocalized in both *avl *and *Rab5 *mutants [15]. Whether Crb or aPKC is the key to the formation of *avl *or *Rab5 *neoplasias, clearly an intimate relationship exists between the different neoplastic tumour suppressors and the polarity complex proteins. Understanding the mechanistic links between these different proteins is therefore required.

### The role of JNK signalling in cooperative neoplastic overgrowth

Our studies confirm previous studies with respect to the key role for JNK in mediating the cooperative neoplastic overgrowth of *scrib *mutants with Ras^ACT ^[16,17]. Oncogenic signals subvert a protective apoptotic JNK response, to an invasive neoplasia. Two identified JNK targets in *scrib*^- ^+ Ras^ACT ^tumours are the matrix metalloproteinase protein, Mmp1 [our unpublished observations, [16,18]], and the integrin-associated scaffolding protein, Paxillin (this study). Mmp expression is required for the tumour invasion since blocking its activity through the expression of Timp (Tissue inhibitor of metalloproteases) restrained *scrib*^- ^+ Ras^ACT ^tumour cells from fusing with and invading the brain lobes but did not abrogate tumour overgrowth or restore pupal development [our unpublished observations, [16,18]]. Both Mmp1 and Pax were induced by JNK signalling, independent of both *scrib *or Ras, since clones of cells expressing an activated allele of the *Drosophila *JNKK homologue, *hemipterous *(Hep^ACT^), also showed strong up-regulation of Pax (see Additional file 5, panel F) and Mmp1 (data not shown). However, it is also likely that Ras and N synergize with JNK to drive expression of novel target genes since, in *scrib *mutants kept alive with P35, JNK remains activated but this does not recapitulate the oncogenic effects of Ras or N [2]. One possible key to the ability of JNK to promote overgrowth in combination with Ras^ACT ^is through blocking differentiation, since expressing Bsk^DN ^in *scrib*^- ^+ Ras^ACT ^tumours restored Ras-induced differentiation and thus restrained tumour over-proliferation enabling pupation of the larvae. However, blocking JNK signalling in *scrib*^- ^+ N^ACT ^tumours could also restore pupation to the tumour-bearing larvae despite massive overgrowth of undifferentiated tumour cells with severely altered cell morphology. Therefore, benign tumour overgrowth is not in itself sufficient to prevent pupation and so synergistic targets of JNK with Ras or N must be responsible. The ability to repress pupation appears to be a property shared by all neoplastic overgrowth in *Drosophila *[38], although the contribution that JNK plays to this in other contexts is not yet known.

Many different cell polarity mutants apart from *scrib *share in the capacity to cooperate with Ras^ACT ^in neoplastic transformation through JNK signalling, including genes that genetically act in opposition to *scrib *such as *sdt *[17] and *crb *(our unpublished observation). This is consistent with JNK being activated indirectly as a consequence of disturbed cell polarity/morphology and further suggests that JNK alone might be sufficient for cooperation. Indeed, co-expression of Hep with Ras^ACT ^has been shown to result in invasive neoplasia [16]. Our results are consistent with this since the cell morphology and proliferative defects of *scrib *mutant clones are rescued by aPKC^CAAXDN^, however, the mutant cells still undergo JNK-mediated apoptosis and can still cooperate with Ras^ACT ^in tumourigeneis. As neoplastic cells between the brain lobes have an elongated mesenchymal-like appearance, JNK and Ras may promote an epithelial-to-mesenchymal transition by impacting on cell shape and/or cell fate pathways irrespective of the loss of Scrib or the blockade in aPKC activity mediated by the dominant negative aPKC transgene.

While JNK is clearly an essential component to neoplastic transformation, the level of JNK activation appears to be critical. Ectopic expression of Ras^ACT ^alone in clones may induce some JNK activation, as judged by the expression of *msn-lacZ*, but is clearly not sufficient to cause neoplastic tumours. In contrast, co-expressing Hep^ACT ^with Ras^ACT ^inefficiently results in neoplastic transformation, presumably because the levels of JNK signalling are too high and this restrains overgrowth or leads to cell death [16]. A loss of Scrib appears to contribute a level of JNK activity strong enough to result in either cell death or neoplastic transformation in cooperation with Ras^ACT^.

### The role of scrib in cooperative neoplastic overgrowth

The analysis of Ras^ACT^-driven tumourigeneisis suggests that JNK activation is both necessary and sufficient for Ras^ACT ^cooperation. However, the fact that blocking aPKC signalling in *scrib*^- ^+ N^ACT ^tumours retards tumour overgrowth suggests that loss of Scrib can also contribute an aPKC-dependent increase in either cell proliferation or cell survival that can profoundly influence the rate of tumour overgrowth. Ras-driven tumours also showed a slight retardation in tumour development with the addition of aPKC^CAAXDN^, although this was much less striking than the effects with N. As JNK signalling remains activated in *scrib *mutant clones expressing aPKC^CAAXDN^, it is likely that JNK can restrain N^ACT^-driven tumour overgrowth, and Ras^ACT ^is more effective than N^ACT ^at counteracting such a JNK-mediated restraint. The aPKC-dependent effects on CycE and increased cell proliferation in *scrib *mutants could help overcome this restraint. Further analysis will be required in order to elucidate the mechanisms involved.

## Conclusion

These results demonstrate distinct aPKC and JNK-dependent pathways through which loss of Scrib promotes tumourigenesis in *Drosophila*. aPKC signalling in *scrib *mutants promotes loss of cell polarity and proliferation, while JNK can either restrain tumour development through cell death or, in cooperation with Ras^ACT ^or N^ACT^, promote aggressive neoplastic tumour overgrowth.

Growing evidence links increased levels of aPKC with the development of human cancers [13,39] and accumulating data support a role for human Scrib in restraining carcinogenesis [reviewed in [7]]. Furthermore, the knockdown of human Scrib in MCF10A cells has recently been shown to cooperate with Ras^ACT ^or Myc in promoting transformation. In the case of Ras^ACT ^expression with Scrib knockdown, cells grown in three-dimensional (3D) culture failed to form the normal polarized acini structures with a central luminal and, instead, adopted a highly invasive morphology [5]. Cooperation with Ras^ACT ^was linked to the ability of Scrib knockdown to potentiate MAPK signalling [5], however, phospho-JNK levels were also increased. JNK signalling is increasingly implicated in mammalian carcinogenesis [40-42], although, as in *Drosophila*, its role can be complex as it also promotes tumour regression through cell death in different contexts [43]. In fact, MCF10A cells grown in 3D culture were also used to investigate Myc-induced transformation of human Scrib knockdown cells, and, in these experiments, luminal filling resulted from Scrib knockdown blocking Myc-induced JNK-dependent cell death [4]. While this is at odds with our *Drosophila *observations, that loss of Scrib promotes JNK-mediated cell death, JNK activation in *scrib *mutant clones was variable and possibly regulated through interactions with neighbouring wild type cells rather than through a cell autonomous up-regulation in JNK signalling [2,44]. Furthermore, other studies in flies have revealed that the *Drosophila *inhibitor of apoptosis 1 (Diap1) is upregulated in *scrib *mutants [45] and, thus, the loss of Scrib may potentially protect *Drosophila *cells from apoptosis in some contexts. Our own studies have also revealed that a loss of Scrib can promote N^ACT^-driven tumour overgrowth through aPKC-dependent pathways involving either increased cell survival or increased cell proliferation. Clearly, further work is required to determine how closely related the tumour suppressor function of Scrib in flies is to its mammalian counterpart. Nevertheless, despite undoubted differences that will exist between the *Drosophila *and the mammalian systems, studies in both organisms have the potential to allow important insights into how the outcome of oncogenic stimulus can be profoundly affected by perturbations in cell polarity networks.

## Abbreviations

aPKC: atypical protein kinase C; Avl: avalanche; Baz: bazooka; BrdU: bromodeoxyuridine; Bsk: basket; Crb: crumbs; CycE: cyclin E; Diap1: Drosophila inhibitor of apoptosis 1; Dlg: discs large; DN: dominant negative; Hep: hemipterous; JNK: Jun N-terminal kinase; Lgl: lethal giant larvae; MARCM: mosaic analysis with repressible marker; Mmp1: matrix metalloproteinase 1; MF: morphogenetic furrow; Msn: misshapen; N: notch; Pax: paxillin; PBS: phosphate-buffered saline; Scrib: scribble; Sdt: stardust.

## Authors' contributions

GRL carried out experiments investigating the role of JNK. KRG assisted with the setting up of experiments and in the preparation of the manuscript. NA carried out experiments with aPKC^ΔN^. HER assisted in the interpretation of experiments and contributed editorial guidance. AMB conceived of the study, designed and carried out experiments, collected and interpreted the data, and wrote the paper. All authors read and approved of the final manuscript.

## Supplementary Material

Additional file 1 ***scrib *mutant cells are eliminated by JNK-dependent apoptosis**. *eyFLP*-induced MARCM clones expressing mCD8-GFP (green). TUNEL detection marks apoptotic cells (grey scale in A-C, red in G), and β-Gal detects *msn-lacZ *enhancer trap activity (grey scale in D-G). A white bar indicates the location of the MF. (A) *FRT82B*. In control mosaic eye discs, TUNEL positive cells are most prominently observed in the eye disc just posterior and anterior of the MF. (B) *FRT82B scrib*^1^. In *scrib *mutant mosaic eye discs, the normal pattern of cell death is largely disrupted and TUNEL positive cells are observed both within (arrow) and surrounding (arrowhead) the GFP positive mutant clones. (C) *FRT82B scrib*^1 ^*UAS-bsk*^*DN*^. *scrib *mutant clones expressing Bsk^DN ^contain very few TUNEL positive cells, although dying cells are still observed in wild-type tissue adjacent to the mutant tissue (arrow). (D) *msn*^06946 ^*FRT82B*. Control mosaic eye discs show low-level expression of *msn-lacZ *posterior to the MF. (E) *msn*^06946 ^*FRT82B scrib*^1^. In *scrib *mutant clones, *msn-lacZ *is ectopically expressed in some patches of mutant tissue (arrow) and in some wild type cells bordering the mutant clones. (F) *msn*^06946 ^*FRT82B scrib*^1 ^*UAS-bsk*^*DN*^. Expressing Bsk^DN ^in *scrib *mutant clones completely abrogates the activation of *msn-lacZ *in the mutant clones, although *msn-lacZ *expressing cells are still sometimes observed in the wild-type tissue adjacent to the mutant tissue (arrow). (G) *msn*^06946 ^*FRT82B scrib*^1^. In *scrib *mutant clones, TUNEL positive and *msn-lacZ *positive cells do not generally overlap, although occasional cells (arrow) express both markers. Click here for file

Additional file 2 **Ectopic Crb disrupts cell morphology and induces JNK-dependent apoptosis and proliferation**. *eyFLP*-induced MARCM clones (green). Grey scale is Elav (A, C, E), phalloidin to mark F-actin (B, D) and BrdU (F). A white bar marks the location of the MF. (A-B) *FRT82B crb*^11*A*22^. *crb *mutant clones show no defects in the pattern of differentiation in the eye disc (A) and no alterations to the normal columnar epithelial cell morphology (B). (C, D) *UAS-crb*^*wt*2*e*^; *FRT82B*. Crb-expressing clones are under-represented relative to the surrounding non-clonal tissue and mildly disrupt the normal pattern of photoreceptor differentiation (C) and the normal columnar cell morphology resulting in cells being excluded from the epithelium (D). (E, F) *UAS-crb*^*wt*2*e*^; *FRT82B UAS-bsk*^*DN*^. Co-expression of Bsk^DN ^with Crb disrupts the normal pattern of differentiation (E), and results in large clones of mutant cells that ectopically proliferate posterior to the MF (F). Click here for file

Additional file 3 **Ectopic expression of activated aPKC disrupts cell morphology and results in ectopic cell proliferation**. *eyFLP*-induced MARCM clones (green). Grey scale is Elav (A, B, E-G) and BrdU (C, D, H, I). Phalloidin marks F-actin in red (A, B, E-G). A white bar indicates the location of the MF. (A-D) *FRT82B UAS-DaPKC*^ΔN^. Ectopic expression of aPKC^ΔN ^in clones results in reduced amounts of clonal tissue that is mostly excluded basally from the epithelium and does not express Elav (A, B) and does not noticeably over-proliferate (C-D), although any proliferative defects are likely to be masked by cell death. (E-I) *FRT82B UAS-DaPKC*^ΔN ^*UAS-bsk*^*DN*^. The co-expression of Bsk^DN ^with aPKC^ΔN ^rescues the small clone phenotype of aPKC^ΔN ^clones alone and most of the mutant tissue has aberrant cell morphology and is extruded basally to form large masses of undifferentiated tissue beneath the dorsal and ventral sides of the eye disc epithelium (E-G). The mutant cells ectopically proliferate posterior to the MF, but not within the MF, in both apical and basal sections (H, I). Click here for file

Additional file 4 **JNK^DN ^represses *scrib*^1 ^+ Ras^ACT ^tumour overgrowth and invasion**. Pairs of larval eye/antennal imaginal discs attached to brain lobes (bl) containing *eyFLP*-induced MARCM clones (green) at day 5 (A, B, F, G), day 7 (C, D) and day 8 (E). Grey scale is Elav (A, F). Red is phalloidin to mark F-actin. (A-B) *UAS-dRas1*^*V*12^; *FRT82B*. Ras^ACT^-expressing clones do not massively overgrow and mutant cells are not observed between the brain lobes. Note the F-actin rich cables (arrows) extending from between the eye/antennal disc to the region between the brain lobes. (C-D) *UAS-dRas1*^*V*12^; *FRT82B scrib*^1^. *scrib*^1 ^+ Ras^ACT ^tumours massively overgrow by day 7 and tumour cells appear to migrate between the brain lobes (arrow in C) along F-actin rich cables (arrow in D). (E) *UAS-dRas1*^*V*12^; *FRT82B crb*^11*A*22 ^*scrib*^1^. Loss of *crb *does not abrogate *scrib*^1 ^+ Ras^ACT ^tumour overgrowth. (F, G) *UAS-dRas1*^*V*12^; *FRT82B scrib*^1 ^*UAS-bsk*^*DN*^. Expression of Bsk^DN ^in *scrib*^1 ^+ Ras^ACT ^tumours prevents tumour overgrowth throughout an extended larval stage of development and blocks invasion of tumour cells between the brain lobes. Click here for file

Additional file 5 **Paxillin is a downstream target of JNK signalling**. Larval eye/antennal imaginal discs containing *eyFLP*-induced MARCM clones (green), with attached brain lobes (bl) in (E). Grey scale is Paxillin. (A) *FRT82B scrib*^1^. *scrib *mutant clones have elevated levels of Paxillin in some mutant cells (arrow). (B) *FRT82B UAS-bsk*^*DN*^. Ectopic expression of Bsk^DN ^in clones does not alter Paxillin levels in the eye disc. (C) *FRT82B scrib*^1 ^*UAS-bsk*^*DN*^. *scrib *mutant clones expressing Bsk^DN ^no longer show elevated levels of Paxillin, although non-clonal tissue adjacent to mutant clones sometimes does ectopically express Paxillin (arrow), presumably reflecting JNK activation in the non-mutant tissue. (D) *UAS-dRas1*^*V*12^; *FRT82B*. Ectopic expression of Ras^ACT ^in clones does not generally elevate Paxillin levels. (E) *UAS-dRas1*^*V*12^; *FRT82B scrib*^1^. Paxillin levels are increased in *scrib*^1 ^+ Ras^ACT ^tumours, most notably at the invasive front, as shown by tumour cells invading into the brain lobes. (F) *UAS-hep*^*ACT*^; *FRT82B UAS-P35*. Clones of tissue expressing an activated allele of the *Drosophila *JNKK homologue, *hemipterous*, Hep (Hep^ACT^), kept alive through the co-expression of the caspase inhibitor P35, ectopically express elevated levels of Paxillin. Click here for file

Additional file 6 **JNK signalling is activated in *scrib*^1 ^+ Ras^ACT ^tumour cells**. Larval eye/antennal imaginal discs containing *eyFLP*-induced MARCM clones (green) at day 5 (A-C, E) and at approximately day 7 (D). A pair of eye discs attached to the brain lobes (bl) is shown in (D). Grey scale is β-Gal to detect *msn-lacZ *enhancer trap activity. Red is phalloidin to mark F-actin. (A) *UAS-dRas1*^*V*12^; *msn*^06946 ^*FRT82B*. Ras^ACT^-expressing clones show a mild increase in *msn-lacZ *reporter activity in eye disc clones, especially in the central region of the disc. (B-D) *UAS-dRas1*^*V*12^; *msn*^06946 ^*FRT82B scrib*^1^. *scrib*^1 ^+ Ras^ACT ^tumours express elevated levels of the *msn-lacZ *enhancer trap, most prominently in cells located basally within the eye disc (B compared to C) and in the region between the eye and antennal (ant) discs (arrow in B). Tumour cells appear to migrate between the brain lobes and ectopically express the *msn-lacZ *enhancer trap (D). (E) *UAS-dRas1*^*V*12^; *msn*^06946 ^*FRT82B scrib*^1 ^*UAS-bsk*^*DN*^. Expression of Bsk^DN ^in *scrib*^1 ^+ Ras^ACT ^tumours prevents *msn-lacZ *expression within the tumour cells. Click here for file

## References

[B1] Bilder D, Li M, Perrimon N (2000). Cooperative regulation of cell polarity and growth by Drosophila tumor suppressors. Science.

[B2] Brumby AM, Richardson HE (2003). scribble mutants cooperate with oncogenic Ras or Notch to cause neoplastic overgrowth in Drosophila. Embo J.

[B3] Pagliarini RA, Xu T (2003). A genetic screen in Drosophila for metastatic behavior. Science.

[B4] Zhan L, Rosenberg A, Bergami KC, Yu M, Xuan Z, Jaffe AB, Allred C, Muthuswamy SK (2008). Deregulation of scribble promotes mammary tumorigenesis and reveals a role for cell polarity in carcinoma. Cell.

[B5] Dow LE, Elsum IA, King CL, Kinross KM, Richardson HE, Humbert PO (2008). Loss of human Scribble cooperates with H-Ras to promote cell invasion through deregulation of MAPK signalling. Oncogene.

[B6] Brumby AM, Richardson HE (2005). Using Drosophila melanogaster to map human cancer pathways. Nat Rev Cancer.

[B7] Humbert PO, Grzeschik NA, Brumby AM, Galea R, Elsum I, Richardson HE (2008). Control of tumourigenesis by the Scribble/Dlg/Lgl polarity module. Oncogene.

[B8] Bilder D, Schober M, Perrimon N (2003). Integrated activity of PDZ protein complexes regulates epithelial polarity. Nat Cell Biol.

[B9] Tanentzapf G, Tepass U (2003). Interactions between the crumbs, lethal giant larvae and bazooka pathways in epithelial polarization. Nat Cell Biol.

[B10] Betschinger J, Mechtler K, Knoblich JA (2003). The Par complex directs asymmetric cell division by phosphorylating the cytoskeletal protein Lgl. Nature.

[B11] Zeitler J, Hsu CP, Dionne H, Bilder D (2004). Domains controlling cell polarity and proliferation in the Drosophila tumor suppressor Scribble. J Cell Biol.

[B12] Rolls MM, Albertson R, Shih HP, Lee CY, Doe CQ (2003). Drosophila aPKC regulates cell polarity and cell proliferation in neuroblasts and epithelia. J Cell Biol.

[B13] Eder AM, Sui X, Rosen DG, Nolden LK, Cheng KW, Lahad JP, Kango-Singh M, Lu KH, Warneke CL, Atkinson EN (2005). Atypical PKCiota contributes to poor prognosis through loss of apical-basal polarity and cyclin E overexpression in ovarian cancer. Proceedings of the National Academy of Sciences of the United States of America.

[B14] Sotillos S, Diaz-Meco MT, Caminero E, Moscat J, Campuzano S (2004). DaPKC-dependent phosphorylation of Crumbs is required for epithelial cell polarity in Drosophila. J Cell Biol.

[B15] Lu H, Bilder D (2005). Endocytic control of epithelial polarity and proliferation in Drosophila. Nat Cell Biol.

[B16] Uhlirova M, Bohmann D (2006). JNK- and Fos-regulated Mmp1 expression cooperates with Ras to induce invasive tumors in Drosophila. Embo J.

[B17] Igaki T, Pagliarini RA, Xu T (2006). Loss of cell polarity drives tumor growth and invasion through JNK activation in Drosophila. Curr Biol.

[B18] Srivastava A, Pastor-Pareja JC, Igaki T, Pagliarini R, Xu T (2007). Basement membrane remodeling is essential for Drosophila disc eversion and tumor invasion. Proceedings of the National Academy of Sciences of the United States of America.

[B19] Lee T, Luo L (1999). Mosaic analysis with a repressible cell marker for studies of gene function in neuronal morphogenesis. Neuron.

[B20] Lee JD, Treisman JE (2001). The role of Wingless signaling in establishing the anteroposterior and dorsoventral axes of the eye disc. Development.

[B21] Spradling AC, Stern D, Beaton A, Rhem EJ, Laverty T, Mozden N, Misra S, Rubin GM (1999). The Berkeley Drosophila Genome Project gene disruption project: Single P-element insertions mutating 25% of vital Drosophila genes. Genetics.

[B22] Bilder D, Perrimon N (2000). Localization of apical epithelial determinants by the basolateral PDZ protein Scribble. Nature.

[B23] Hay BA, Wolff T, Rubin GM (1994). Expression of baculovirus P35 prevents cell death in Drosophila. Development.

[B24] Adachi-Yamada T, Nakamura M, Irie K, Tomoyasu Y, Sano Y, Mori E, Goto S, Ueno N, Nishida Y, Matsumoto K (1999). p38 mitogen-activated protein kinase can be involved in transforming growth factor beta superfamily signal transduction in Drosophila wing morphogenesis. Mol Cell Biol.

[B25] Tepass U, Theres C, Knust E (1990). crumbs encodes an EGF-like protein expressed on apical membranes of Drosophila epithelial cells and required for organization of epithelia. Cell.

[B26] Wodarz A, Hinz U, Engelbert M, Knust E (1995). Expression of crumbs confers apical character on plasma membrane domains of ectodermal epithelia of Drosophila. Cell.

[B27] Karim FD, Rubin GM (1998). Ectopic expression of activated Ras1 induces hyperplastic growth and increased cell death in Drosophila imaginal tissues. Development.

[B28] Go MJ, Eastman DS, Artavanis-Tsakonas S (1998). Cell proliferation control by Notch signaling in Drosophila development. Development.

[B29] Yagi R, Ishimaru S, Yano H, Gaul U, Hanafusa H, Sabe H (2001). A novel muscle LIM-only protein is generated from the paxillin gene locus in Drosophila. EMBO Rep.

[B30] Uhlirova M, Jasper H, Bohmann D (2005). Non-cell-autonomous induction of tissue overgrowth by JNK/Ras cooperation in a Drosophila tumor model. Proceedings of the National Academy of Sciences of the United States of America.

[B31] Mattila J, Omelyanchuk L, Kyttala S, Turunen H, Nokkala S (2005). Role of Jun N-terminal Kinase (JNK) signaling in the wound healing and regeneration of a Drosophila melanogaster wing imaginal disc. Int J Dev Biol.

[B32] Izaddoost S, Nam SC, Bhat MA, Bellen HJ, Choi KW (2002). Drosophila Crumbs is a positional cue in photoreceptor adherens junctions and rhabdomeres. Nature.

[B33] Pellikka M, Tanentzapf G, Pinto M, Smith C, McGlade CJ, Ready DF, Tepass U (2002). Crumbs, the Drosophila homologue of human CRB1/RP12, is essential for photoreceptor morphogenesis. Nature.

[B34] Fan Y, Bergmann A (2008). Apoptosis-induced compensatory proliferation. The Cell is dead. Long live the Cell!. Trends Cell Biol.

[B35] Wirtz-Peitz F, Nishimura T, Knoblich JA (2008). Linking cell cycle to asymmetric division: Aurora-A phosphorylates the Par complex to regulate Numb localization. Cell.

[B36] Osmani N, Vitale N, Borg JP, Etienne-Manneville S (2006). Scrib controls Cdc42 localization and activity to promote cell polarization during astrocyte migration. Curr Biol.

[B37] Dow LE, Kauffman JS, Caddy J, Zarbalis K, Peterson AS, Jane SM, Russell SM, Humbert PO (2007). The tumour-suppressor Scribble dictates cell polarity during directed epithelial migration: regulation of Rho GTPase recruitment to the leading edge. Oncogene.

[B38] Menut L, Vaccari T, Dionne H, Hill J, Wu G, Bilder D (2007). A mosaic genetic screen for Drosophila neoplastic tumor suppressor genes based on defective pupation. Genetics.

[B39] Regala RP, Weems C, Jamieson L, Copland JA, Thompson EA, Fields AP (2005). Atypical protein kinase Ciota plays a critical role in human lung cancer cell growth and tumorigenicity. The Journal of biological chemistry.

[B40] Shibata W, Maeda S, Hikiba Y, Yanai A, Sakamoto K, Nakagawa H, Ogura K, Karin M, Omata M (2008). c-Jun NH2-terminal kinase 1 is a critical regulator for the development of gastric cancer in mice. Cancer Res.

[B41] Nielsen C, Thastrup J, Bottzauw T, Jaattela M, Kallunki T (2007). c-Jun NH2-terminal kinase 2 is required for Ras transformation independently of activator protein 1. Cancer Res.

[B42] Zhang JY, Adams AE, Ridky TW, Tao S, Khavari PA (2007). Tumor necrosis factor receptor 1/c-Jun-NH2-kinase signaling promotes human neoplasia. Cancer Res.

[B43] Weston CR, Davis RJ (2007). The JNK signal transduction pathway. Curr Opin Cell Biol.

[B44] Igaki T, Pastor-Pareja JC, Aonuma H, Miura M, Xu T (2009). Intrinsic tumor suppression and epithelial maintenance by endocytic activation of Eiger/TNF signaling in Drosophila. Developmental cell.

[B45] Zhao M, Szafranski P, Hall CA, Goode S (2008). Basolateral junctions utilize warts signaling to control epithelial-mesenchymal transition and proliferation crucial for migration and invasion of Drosophila ovarian epithelial cells. Genetics.

