# Supplementary figures and images for: scribble mutants promote aPKC and JNK-dependent epithelial neoplasia independently of Crumbs
Source: BMC Biol. 2009 Sep 24;7:62. doi: 10.1186/1741-7007-7-62 (PMC2760524; doi:10.1186/1741-7007-7-62)

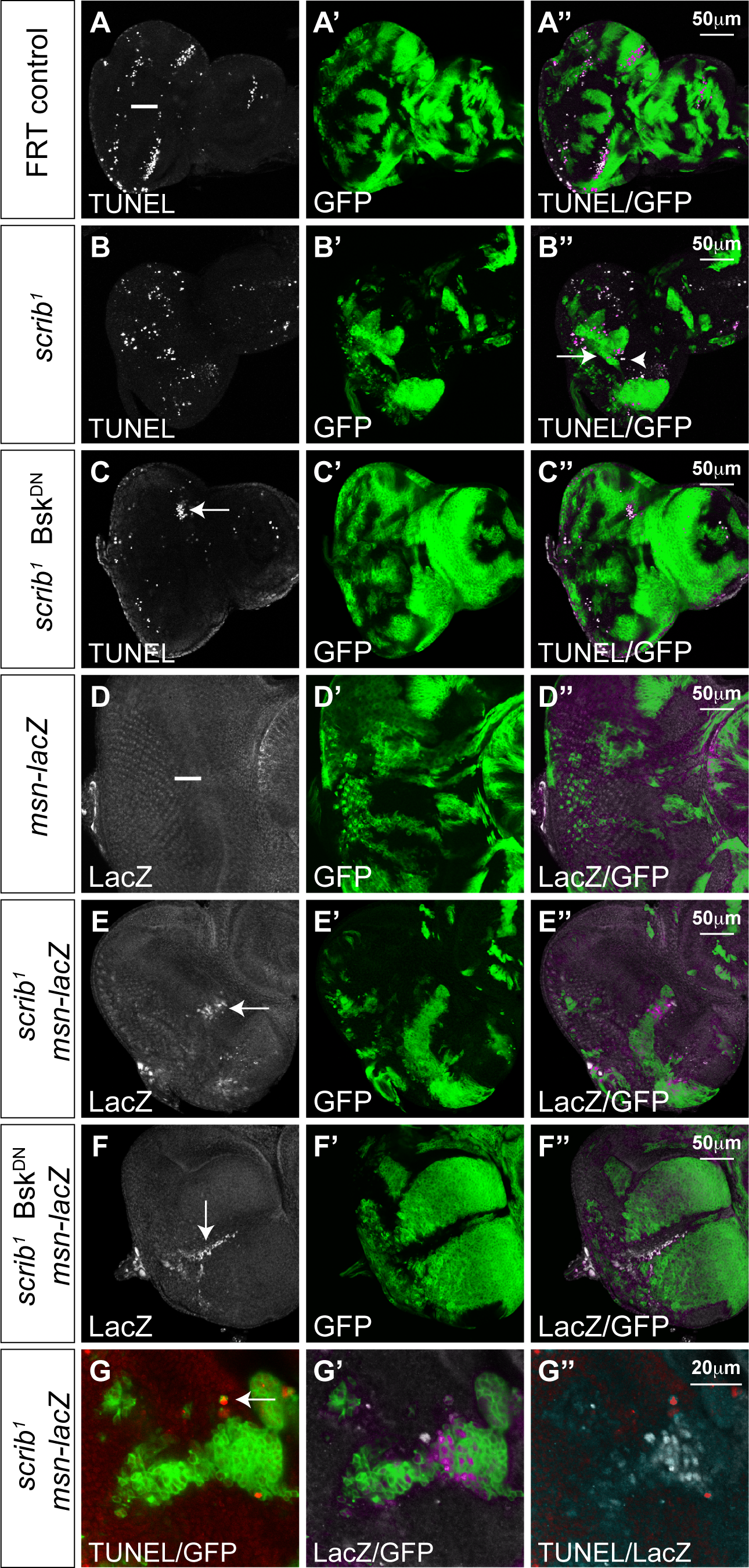

Supplement: Additional file 1 — scrib mutant cells are eliminated by JNK-dependent apoptosis. eyFLP-induced MARCM clones expressing mCD8-GFP (green). TUNEL detection marks apoptotic cells (grey scale in A-C, red in G), and β-Gal detects msn-lacZ enhancer trap activity (grey scale in D-G). A white bar indicates the location of the MF. (A) FRT82B. In control mosaic eye discs, TUNEL positive cells are most prominently observed in the eye disc just posterior and anterior of the MF. (B) FRT82B scrib1. In scrib mutant mosaic eye discs, the normal pattern of cell death is largely disrupted and TUNEL positive cells are observed both within (arrow) and surrounding (arrowhead) the GFP positive mutant clones. (C) FRT82B scrib1 UAS-bskDN. scrib mutant clones expressing BskDN contain very few TUNEL positive cells, although dying cells are still observed in wild-type tissue adjacent to the mutant tissue (arrow). (D) msn06946 FRT82B. Control mosaic eye discs show low-level expression of msn-lacZ posterior to the MF. (E) msn06946 FRT82B scrib1. In scrib mutant clones, msn-lacZ is ectopically expressed in some patches of mutant tissue (arrow) and in some wild type cells bordering the mutant clones. (F) msn06946 FRT82B scrib1 UAS-bskDN. Expressing BskDN in scrib mutant clones completely abrogates the activation of msn-lacZ in the mutant clones, although msn-lacZ expressing cells are still sometimes observed in the wild-type tissue adjacent to the mutant tissue (arrow). (G) msn06946 FRT82B scrib1. In scrib mutant clones, TUNEL positive and msn-lacZ positive cells do not generally overlap, although occasional cells (arrow) express both markers. [file 1741-7007-7-62-S1.tiff]

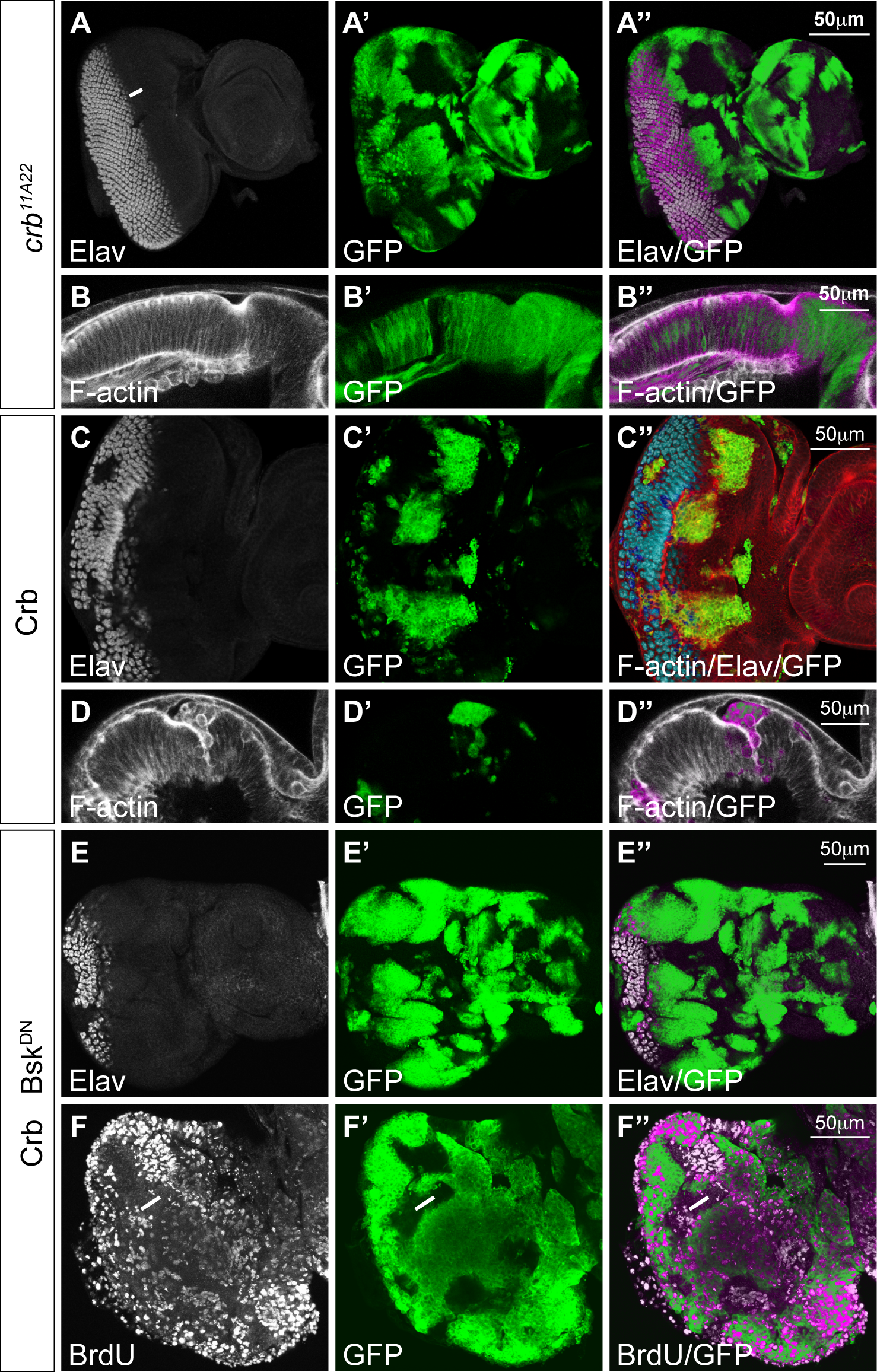

Supplement: Additional file 2 — Ectopic Crb disrupts cell morphology and induces JNK-dependent apoptosis and proliferation. eyFLP-induced MARCM clones (green). Grey scale is Elav (A, C, E), phalloidin to mark F-actin (B, D) and BrdU (F). A white bar marks the location of the MF. (A-B) FRT82B crb11A22. crb mutant clones show no defects in the pattern of differentiation in the eye disc (A) and no alterations to the normal columnar epithelial cell morphology (B). (C, D) UAS-crbwt2e; FRT82B. Crb-expressing clones are under-represented relative to the surrounding non-clonal tissue and mildly disrupt the normal pattern of photoreceptor differentiation (C) and the normal columnar cell morphology resulting in cells being excluded from the epithelium (D). (E, F) UAS-crbwt2e; FRT82B UAS-bskDN. Co-expression of BskDN with Crb disrupts the normal pattern of differentiation (E), and results in large clones of mutant cells that ectopically proliferate posterior to the MF (F). [file 1741-7007-7-62-S2.tiff]

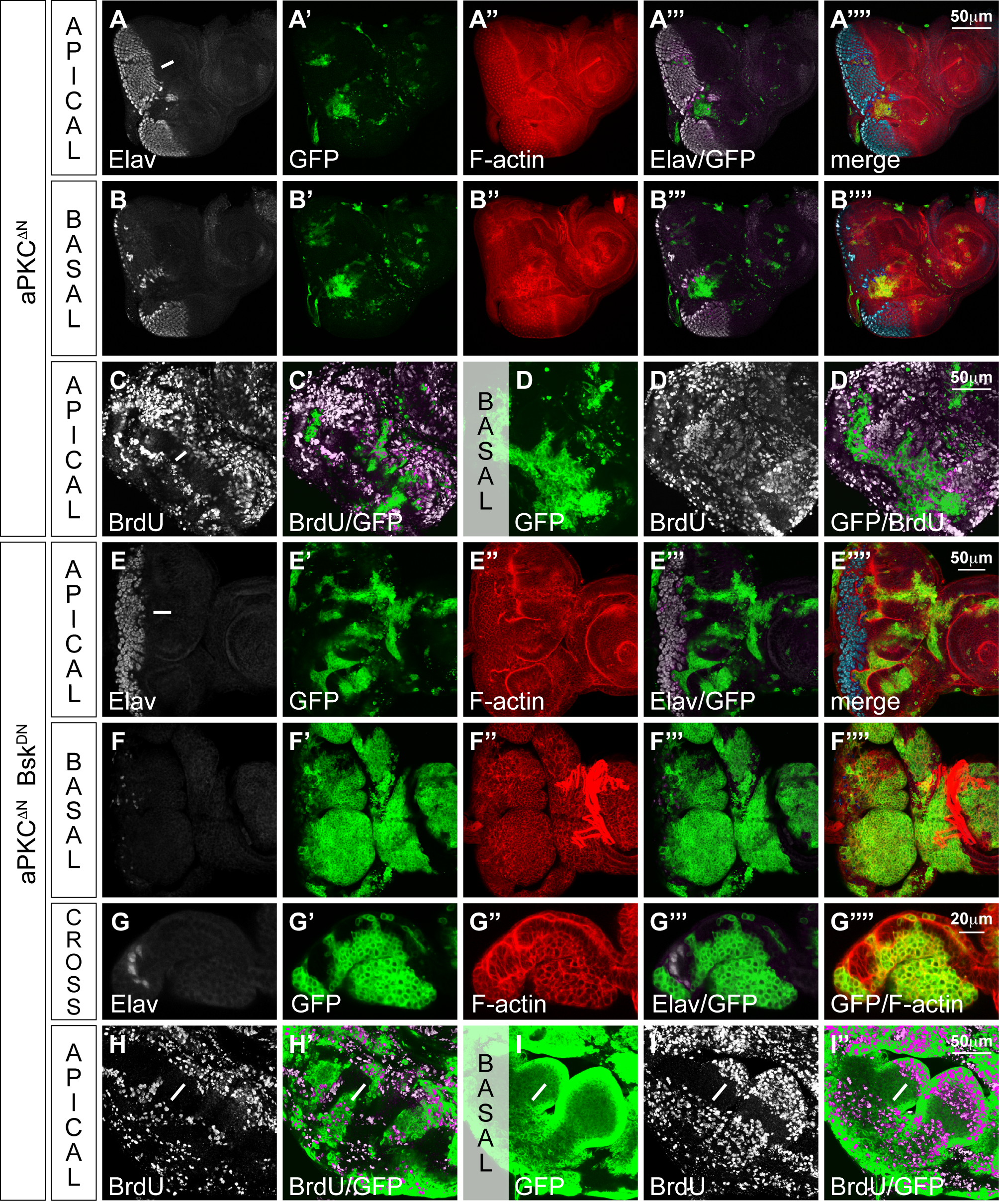

Supplement: Additional file 3 — Ectopic expression of activated aPKC disrupts cell morphology and results in ectopic cell proliferation. eyFLP-induced MARCM clones (green). Grey scale is Elav (A, B, E-G) and BrdU (C, D, H, I). Phalloidin marks F-actin in red (A, B, E-G). A white bar indicates the location of the MF. (A-D) FRT82B UAS-DaPKCΔN. Ectopic expression of aPKCΔN in clones results in reduced amounts of clonal tissue that is mostly excluded basally from the epithelium and does not express Elav (A, B) and does not noticeably over-proliferate (C-D), although any proliferative defects are likely to be masked by cell death. (E-I) FRT82B UAS-DaPKCΔN UAS-bskDN. The co-expression of BskDN with aPKCΔN rescues the small clone phenotype of aPKCΔN clones alone and most of the mutant tissue has aberrant cell morphology and is extruded basally to form large masses of undifferentiated tissue beneath the dorsal and ventral sides of the eye disc epithelium (E-G). The mutant cells ectopically proliferate posterior to the MF, but not within the MF, in both apical and basal sections (H, I). [file 1741-7007-7-62-S3.tiff]

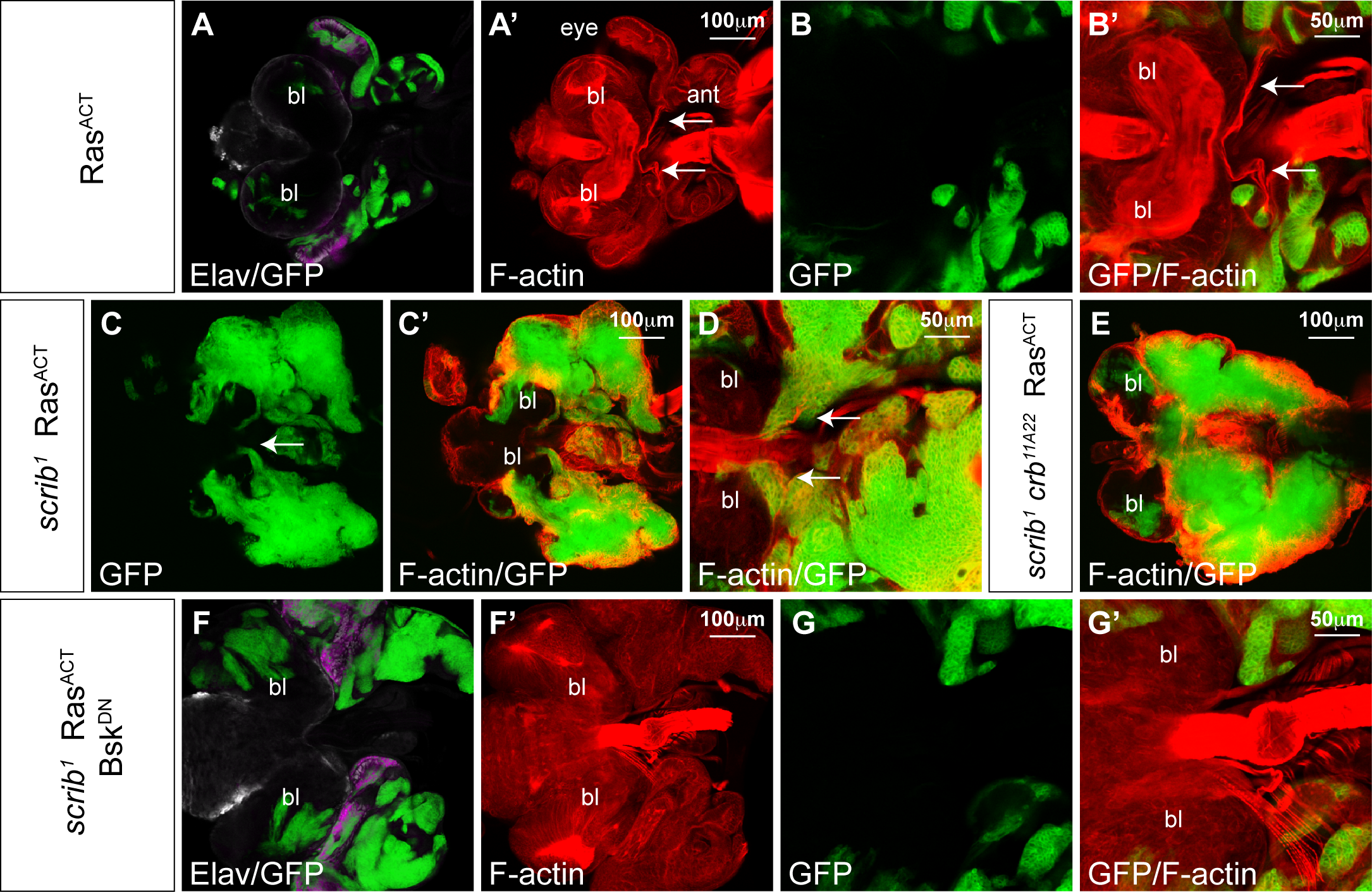

Supplement: Additional file 4 — JNKDN represses scrib1 + RasACT tumour overgrowth and invasion. Pairs of larval eye/antennal imaginal discs attached to brain lobes (bl) containing eyFLP-induced MARCM clones (green) at day 5 (A, B, F, G), day 7 (C, D) and day 8 (E). Grey scale is Elav (A, F). Red is phalloidin to mark F-actin. (A-B) UAS-dRas1V12; FRT82B. RasACT-expressing clones do not massively overgrow and mutant cells are not observed between the brain lobes. Note the F-actin rich cables (arrows) extending from between the eye/antennal disc to the region between the brain lobes. (C-D) UAS-dRas1V12; FRT82B scrib1. scrib1 + RasACT tumours massively overgrow by day 7 and tumour cells appear to migrate between the brain lobes (arrow in C) along F-actin rich cables (arrow in D). (E) UAS-dRas1V12; FRT82B crb11A22 scrib1. Loss of crb does not abrogate scrib1 + RasACT tumour overgrowth. (F, G) UAS-dRas1V12; FRT82B scrib1 UAS-bskDN. Expression of BskDN in scrib1 + RasACT tumours prevents tumour overgrowth throughout an extended larval stage of development and blocks invasion of tumour cells between the brain lobes. [file 1741-7007-7-62-S4.tiff]

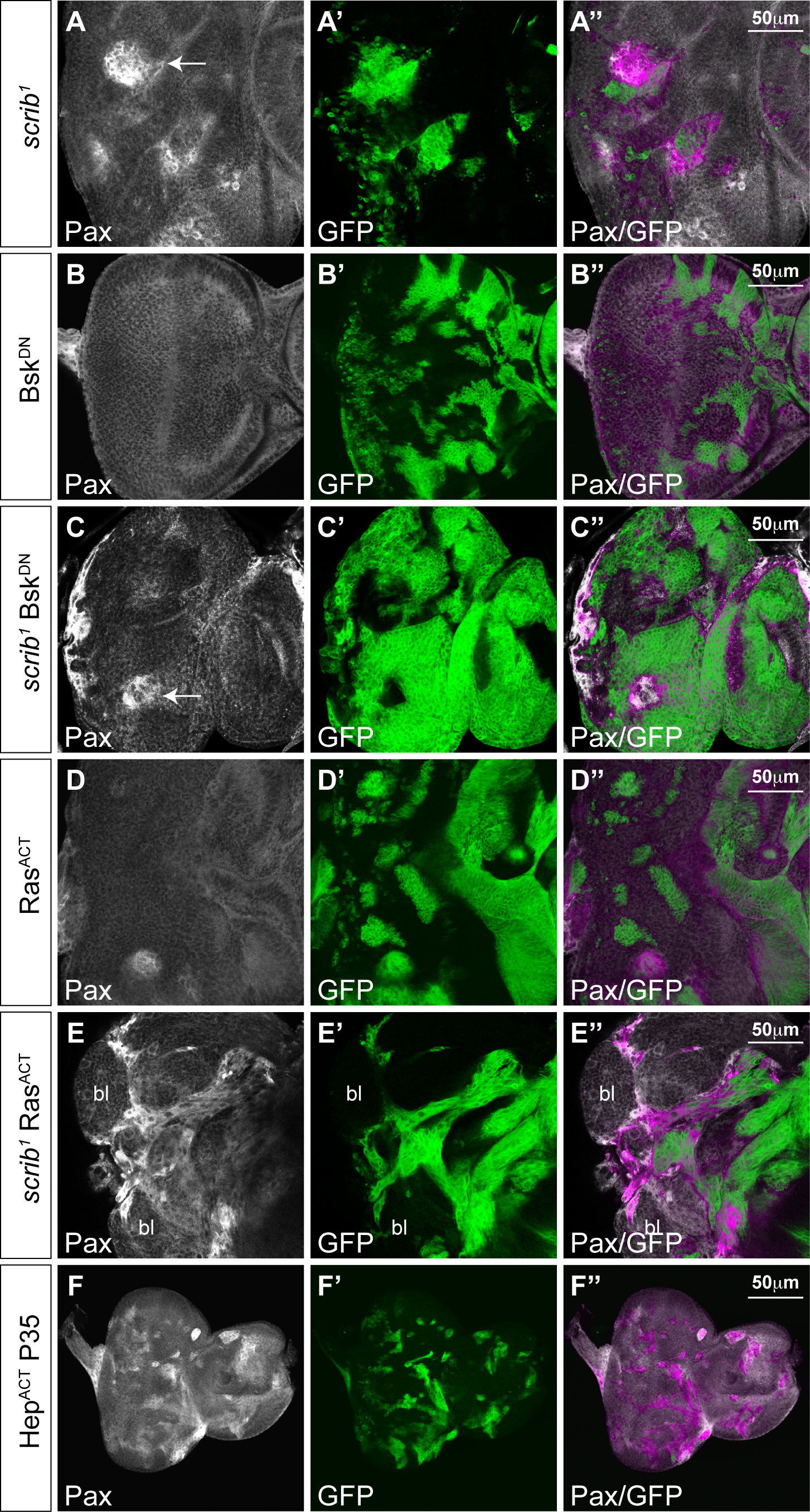

Supplement: Additional file 5 — Paxillin is a downstream target of JNK signalling. Larval eye/antennal imaginal discs containing eyFLP-induced MARCM clones (green), with attached brain lobes (bl) in (E). Grey scale is Paxillin. (A) FRT82B scrib1. scrib mutant clones have elevated levels of Paxillin in some mutant cells (arrow). (B) FRT82B UAS-bskDN. Ectopic expression of BskDN in clones does not alter Paxillin levels in the eye disc. (C) FRT82B scrib1 UAS-bskDN. scrib mutant clones expressing BskDN no longer show elevated levels of Paxillin, although non-clonal tissue adjacent to mutant clones sometimes does ectopically express Paxillin (arrow), presumably reflecting JNK activation in the non-mutant tissue. (D) UAS-dRas1V12; FRT82B. Ectopic expression of RasACT in clones does not generally elevate Paxillin levels. (E) UAS-dRas1V12; FRT82B scrib1. Paxillin levels are increased in scrib1 + RasACT tumours, most notably at the invasive front, as shown by tumour cells invading into the brain lobes. (F) UAS-hepACT; FRT82B UAS-P35. Clones of tissue expressing an activated allele of the Drosophila JNKK homologue, hemipterous, Hep (HepACT), kept alive through the co-expression of the caspase inhibitor P35, ectopically express elevated levels of Paxillin. [file 1741-7007-7-62-S5.tiff]

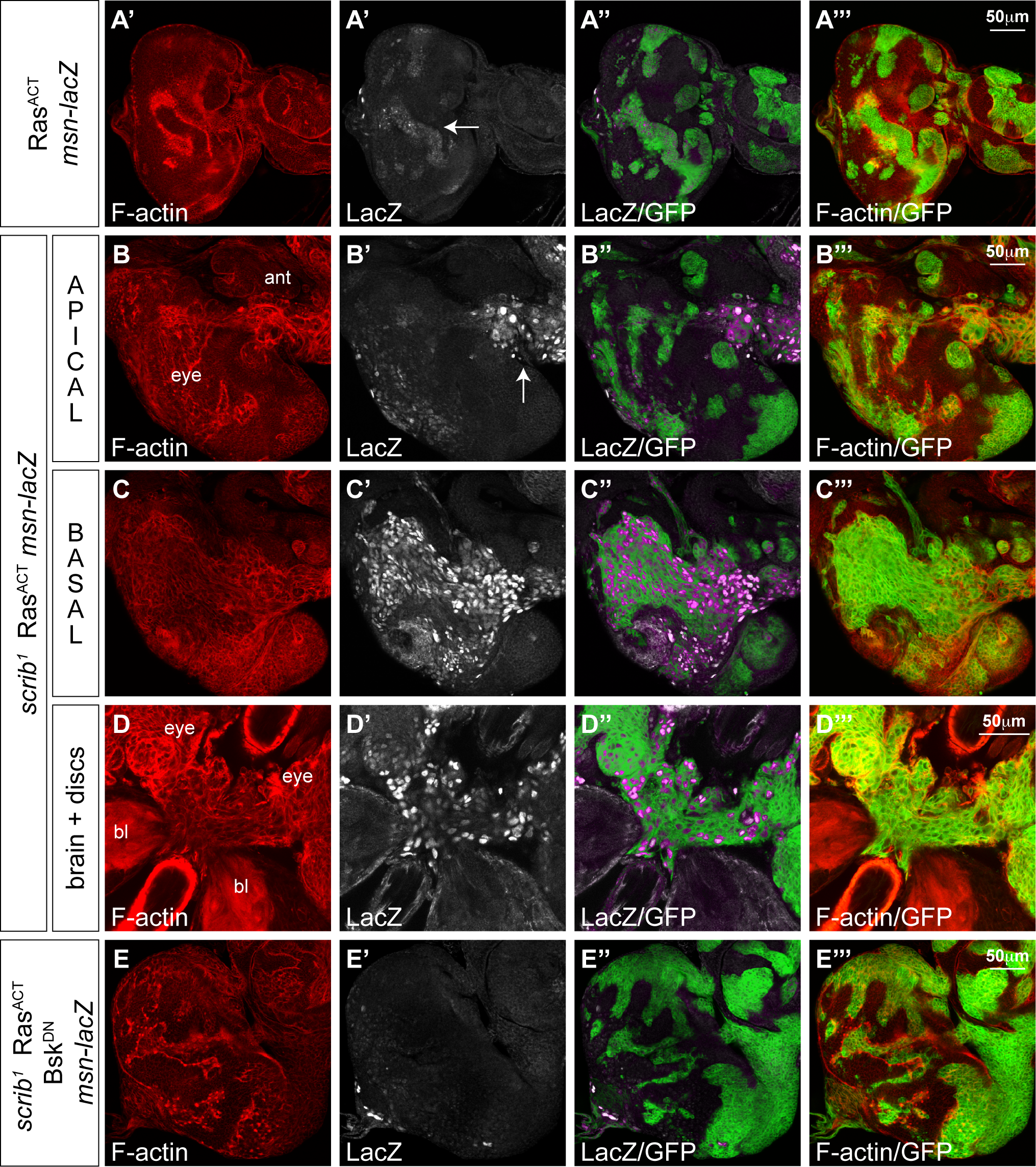

Supplement: Additional file 6 — JNK signalling is activated in scrib1 + RasACT tumour cells. Larval eye/antennal imaginal discs containing eyFLP-induced MARCM clones (green) at day 5 (A-C, E) and at approximately day 7 (D). A pair of eye discs attached to the brain lobes (bl) is shown in (D). Grey scale is β-Gal to detect msn-lacZ enhancer trap activity. Red is phalloidin to mark F-actin. (A) UAS-dRas1V12; msn06946 FRT82B. RasACT-expressing clones show a mild increase in msn-lacZ reporter activity in eye disc clones, especially in the central region of the disc. (B-D) UAS-dRas1V12; msn06946 FRT82B scrib1. scrib1 + RasACT tumours express elevated levels of the msn-lacZ enhancer trap, most prominently in cells located basally within the eye disc (B compared to C) and in the region between the eye and antennal (ant) discs (arrow in B). Tumour cells appear to migrate between the brain lobes and ectopically express the msn-lacZ enhancer trap (D). (E) UAS-dRas1V12; msn06946 FRT82B scrib1 UAS-bskDN. Expression of BskDN in scrib1 + RasACT tumours prevents msn-lacZ expression within the tumour cells. [file 1741-7007-7-62-S6.tiff]
